# Application of low-density polyethylene (LDPE) passive samplers for monitoring PAHs in groundwater

**DOI:** 10.1007/s11356-024-34731-7

**Published:** 2024-08-29

**Authors:** Ibukun Ola, Carsten Drebenstedt, Robert M. Burgess, Ian J. Allan, Nils Hoth, Christoph Külls

**Affiliations:** 1 Institute of Mining and Special Civil Engineering, Technical University Mining Academy, Gustav-Zeuner Street 1A, 09599 Freiberg, Germany; 2 U.S. Environmental Protection Agency, Office of Research and Development, Center for Environmental Measurement and Modeling, Atlantic Coastal Environmental Sciences Division, 27 Tarzwell Drive, Narragansett, RI 02882 USA; 3 Norwegian Institute for Water Research (NIVA), Økernveien 94, NO-0579 Oslo, Norway; 4 Labor Für Hydrologie Und Internationale Wasserwirtschaft, Technische Hochschule Lübeck, 23562 Lübeck, Schleswig-Holstein Germany

**Keywords:** Equilibrium passive sampling, Groundwater, Polyethylene, Monitoring wells, Performance reference compounds (PRCs)

## Abstract

Equilibrium passive sampling continues to find increasing use for performing in situ assessments and monitoring of hydrophobic organic compounds (HOCs). Although this method has been successfully used in several field studies including open surface waters and sediments, comparatively, their use in groundwater has been very limited. In this study, low-density polyethylene (LDPE) passive samplers were deployed for 80 days in three groundwater wells contaminated with polycyclic aromatic hydrocarbons (PAHs). Prior to deployment, LDPE was loaded with performance reference compounds (PRCs) consisting of deuterated PAHs and their release used to ascertain system equilibrium. Within the 80-day deployment period, LDPE-groundwater equilibrium was confirmed for PAHs with molecular weights (MWs) in the range of 178 to 228 (i.e. anthracene, chrysene). Measured freely dissolved concentrations (*C*_*w*_) were between one to three orders of magnitude lower than the total filtered concentrations (*C*_total_) in the studied wells. The sum of PAHs (ΣPAHs) measured based on *C*_*w*_ and *C*_total_ were 2.05, 0.07 and 29.2 μg L^−1^ and 197, 59.7 and 1010 μg L^−1^, at wells 1, 2 and 3, respectively. A separate dataset, comprising long-term (2010 to 2022) concentrations of PAHs in total (i.e., unfiltered) groundwater, is also presented to provide insight into PAH contamination levels at the assessed groundwater wells based on conventional measurement. Estimated in situ LDPE daily clearance volumes (2.34 to 27.56 Ld^−1^) for the target analytes were far less than the daily turnover of ground water (144 to 348 Ld^−1^) encountered in the wells eliminating the possibility of depletive sampling of the groundwater by the passive samplers. These results represent the first published study on the practical application of equilibrium passive sampling using LDPE for monitoring and quantitatively assessing PAHs in groundwater. Also, this work demonstrates that LDPEs are a useful tool for measuring the *C*_*w*_ of PAHs in groundwater, a critical contaminant in many ecological and human health risk assessments.

**Supplementary Information:**

The online version contains supplementary material available at 10.1007/s11356-024-34731-7.

## Introduction

Groundwater represents one of the safest sources of drinking water and is, in general, less susceptible to contamination than open surface waters (e.g. lakes, sea, rivers) (Black 2016; Schmoll et al. 2006). This is in part because the rocks and soil through which groundwater flow along with natural processes of degradation limit the amount of any contaminants reaching groundwater aquifers (Morris et al. 2003). However, sometimes, contaminants may find their way into groundwater at concentrations sufficiently high to threaten water supply systems and impact other groundwater-dependent ecosystem services. The remediation of groundwater contamination is often complex, expensive and requires long-term (in order of decades) monitoring of quality improvements and assessment of remediation performance (Obiri-Nyarko et al. 2014). Therefore, reliable and efficient groundwater monitoring approaches are an important early-warning tool for groundwater quality assessment and protection.

Improved methods of contaminant monitoring and risk assessment are still being sought for groundwater systems (Kaserzon et al. 2019; Cornelissen et al. 2009; Bopp et al. 2005). In recent times, passive sampling has been of particular interest because it is a strategy based on time-integrated sampling, provides insights into contaminants bioavailability, is simple to use and with low cost. Typically, passive sampling involves the deployment of an absorbing material allowing for the long-term (days to months) accumulation of target chemicals using the mechanisms of diffusion (Jones et al. 2015; Huckins et al. 2006). Passive sampling can be grouped into two types based on their operating principle: kinetic and equilibrium methods. Kinetic passive sampling often involves the use of mostly high surface area–sorbent materials allowing contaminant uptake to remain within the kinetic regime over a long period of time (Vermeirssen et al. 2012). This type of sampler has been extensively used to monitor relatively hydrophilic groundwater contaminants like hydrophilic pesticides and pharmaceuticals. Examples include polar organic chemical integrative sampler (POCIS) (Berho et al. 2013; Dougherty et al. 2010; Soulier et al. 2016), Chemcatcher (Pinasseau et al. 2020; Ahkola et al. 2017), ceramic dosimeters (Bopp et al. 2005, 2007), passive smart filters (Jonge and Rothenberg 2005) and microporous polyethylene (PE) packed with an anion exchange sorbent—Strata X-AW (Kaserzon et al. 2019). The equilibrium passive sampling approach involves the exposure of primarily hydrophobic polymers (e.g. low-density polyethylene (LDPE), polydimethylsiloxane (PDMS)) to water for sufficient time allowing the target contaminant to attain a thermodynamic equilibrium between the sorbing material and water phase (Vrana et al. 2005a). In addition, performance reference compounds (PRCs) can be used with equilibrium samplers to confirm equilibrium conditions are achieved. Equilibrium samplers are known to sample non-polar HOCs more efficiently; thus, they have been extensively used in the monitoring of these types of compounds (e.g. PCBs, PAHs, chlorinated pesticides) in surface water and sediment pore water (Burgess et al. 2015; Belháčová-Minaříková et al. 2022; Allan et al. 2021; Sobotka et al. 2022). In contrast, their use in groundwater monitoring of non-polar HOCs remains less well-established and very limited. A study by Cornelissen et al. (2009) involving ultrathin (17 μm thickness) polyoxymethylene (POM) strips and two others by (Gustavson and Harkin 2000; Vrana et al. 2005b) using semi-permeable membrane devices (SPMDs) represent the published works on equilibrium passive sampling of HOCs in groundwater.

Given groundwater resources are of high regulatory importance within most legislative environmental frameworks and passive sampling represents a new, potentially powerful tool, it is essential to adequately characterise the performance of passive sampling, including equilibrium passive sampling, in groundwater. This would help provide evidence on the potential valuable role equilibrium passive samplers can play in groundwater quality monitoring and regulatory compliance. To address this need, in this study, the following investigations were carried out: (1) a performance test of LDPE as a viable equilibrium passive sampler for the long-term monitoring of PAHs (i.e. U.S. EPA 16 PAHs) in contaminated groundwater wells and (2) an estimation of the *C*_*w*_ and total dissolved concentrations (*C*_total_) of the target PAHs in three contaminated groundwater wells. In one of the wells, PAH sorption to the PE was measured over time to provide insight into the accumulation dynamics of the target chemicals. All exposed LDPEs were loaded with a series of five PRCs to ascertain the degree of equilibrium reached during deployment and to determine in situ calibration parameters. Building upon our prior laboratory investigation, which centred around deriving polyethylene-water partition coefficients (*K*_PE-W_) for polycyclic aromatic hydrocarbons (PAHs) and analysing PAH partitioning into LDPE, we derived an empirical equation. This equation holds the potential to estimate *K*_PE-W_ values for PAHs and other hydrophobic organic compounds (HOCs) beyond those covered in our original study. Importantly, this equation was employed to estimate *K*_PE-W_ values for the PAHs examined in this current study. To our knowledge, this study represents the first published work on the use of equilibrium LDPE passive sampling to monitor PAHs in groundwater. The *C*_total_ and *C*_*w*_ are two parameters that provide useful information on different parameters: *C*_total_ supplies data relevant for chemical transport modelling while *C*_*w*_ data are useful for determining the bioavailable concentrations of PAHs in groundwater.

## Materials and methods

### Study area description and lithology

The study area (to remain anonymous), located in eastern Germany, overlies a shallow (< 10 m) aquifer in a former mining area now turned into an urban centre. Owing to the long history of mining operations, the soil and groundwater are known to be extensively contaminated with semi-volatile organic chemicals (e.g. PAHs, phenols) as well as organic volatiles including benzene, toluene, ethylbenzene and xylenes BTEX and cyanides. Three wells (denoted as well 1, well 2 and well 3; diameter 12.5 cm each) were studied. Wells 1, 2 and 3 were installed in the years 2007, 1999 and 1993, respectively. In all cases, the wells have between 2 to 3-m screen intervals, that is, a filtered zone where the LDPE samplers were deployed during sampling. The screen intervals are located in native sandy and gravel layer deposits having medium sand to coarse sand and fine gravel to medium gravel consistency. Data on the selected geophysical parameters of the sampling wells are provided in Table S1.

### Chemicals

Solvents used in this study included cyclohexane (99.5% purity; Th. Geyer GmbH & Co. KG, Renningen Germany), methanol and acetone (GC grade; Sigma Aldrich, Steinheim Germany) and deionised water (Sigma Aldrich, Steinheim Germany). Five deuterated PAHs were used as performance reference compounds (PRCs) including d10-anthracene, d10-fluoranthene, d12-benzo(a)anthracene, d12-benzo(a)pyrene and d14-dibenzo(a,h)anthracene. Internal standards (IS) included d10-acenaphthene, d12-chrysene, d8-napthalene, d12-perylene, d10-phenathrene and d10-pyrene all with a purity of > 99%. PRCs and IS were purchased from Neochema GmbH (Bodenheim, Germany).

### Sampling groundwater

Samples (triplicate 3 L per well) were collected through a Tygon® tubing using a submersible pump before LDPE passive samplers were deployed in February 2022. Prior to the collection of water samples, a purging process was performed. It involved the pumping of between one and two sample volumes from the groundwater wells. The purging process was considered necessary to (i) remove standing well water and (ii) equilibrate the pump and tubing with ambient contaminant levels to limit sorptive losses. Samples were stored in 3-L glass bottles, and NaN_3_ (0.1 g L^−1^) was immediately added to minimize bacterial degradation. From each 3-L sample, 30 mL was transferred to Falcon tubes (Brand GmbH & Co. KG, Germany) acidified by adding 300-μL 1 M HCl and used for total organic carbon (TOC) analysis. Separation of particulate organic carbon (POC) and dissolved organic carbon (DOC) was achieved through the filtration of water samples. The applied filtration process protocol is described next.

### Filtration

Filters (0.45 µm pore-size) (Chromafil® Xtra H-PTFE-45/25, Macherey–Nagel) were used to filter the water samples. A fraction (the first 0.5 L) of each filtered sample was discarded to avoid filter sorption issues. Two aliquots (30 mL and 250 mL) were taken from each filtered sample. The first aliquot was used for the DOC analysis, and the second volume was liquid–liquid extracted and applied for measuring the total dissolved water concentration (*C*_total_) of the target contaminants.

### Total and dissolved organic carbon analysis

TOC/DOC were characterised with a TOC-V-CPH analyser (Shimadzu, Kyoto, Japan) with an integrated auto-sampler ASI-V (Shimadzu, Japan) following German standard method DIN EN 1484:^2019^–0 (DIN). For the measurement of organic carbon, the standards used were prepared from potassium hydrogen phthalate in ultra-pure water. Unfiltered water samples were used to report TOC concentrations while filtered water samples (i.e. post-0.45 µm) were operationally defined for making the DOC measurements.

### LDPE preparation

LDPE (70 μm thickness) was obtained from Brentwood Plastics (St. Louis, MO, USA) kindly supplied by K. Booij (PaSOC, The Netherlands). LDPE was resized into several long strips (length = 30 cm, width = 1 cm, mass = 0.21 g). The LDPEs were loaded with PRCs following procedures outlined in Booij et al. (2002). Briefly, LDPE strips were incubated in a PRC-spiked methanol/water 80/20 (V/V) mixture, and the water content was increased in 10% increments (over 18 days mixed at 150 rpm) until a 50/50 methanol/water content was reached. Following the PRC loading process, clean laboratory tissue paper was used to remove traces of methanol from the LDPE strips and three LDPE strips sampled and analysed to determine the initial concentrations of PRCs (*C*_prci_); each LDPE strip contained between 1.32 and 2.64 µg of each PRC (Table S2). The relative standard deviations (RSDs) of the loaded PRCs were between 3.5 and 8.2% except d12-benzo(a)anthracene (19.8%).

For simple placement and stability during LDPE deployment, three support frames (denoted as frames A, B and C) were constructed (Figure S1) from stainless steel 1.4301 V2A sheets (thickness = 2 mm, Stahlog GmbH, Hörselberg-Hainich, Germany). Frames A and B received six series of PRC-spiked LDPE strips each, while frame C received 18 series of PRC-spiked LDPE strips. To ease recovery of frame-supported LDPEs, a 6-mm-thick line was attached to each frame (Figure S1).

### LDPE deployment and sampling

Frame-supported passive LDPE samplers were lowered into three groundwater wells and left in place for 80 days. Frames A and B were placed in well 1 and well 2, respectively, and were sampled twice (42 days and 80 days) while frame C was placed into well 3 and was sampled six times (14 days, 28 days, 42 days, 56 days, 70 days, 80 days). Triplicate PEs were removed on each sampling occasion.

The choice of an 80-day sampling deployment duration was based on several considerations. The study by Cornelissen et al. (2009) indicated that equilibrium passive samplers require sufficient time to reach equilibrium with the groundwater environment to provide reliable measurements of contaminant concentrations. The 80-day duration ensures that the samplers have adequate time to accumulate target analytes to detectable levels, particularly for low-concentration contaminants. Additionally, groundwater systems can exhibit temporal variability in contaminant concentrations, and an extended deployment period allows for the capture of this variability, providing a more comprehensive understanding of contaminant dynamics over time. Furthermore, the 80-day duration strikes a balance between practical field deployment constraints and the need for thorough temporal coverage. It minimizes the frequency of field visits while still enabling multiple sampling points (as evidenced by the intermediate sampling times) to assess changes over the deployment duration.

No fouling was observed on the surface of the retrieved LDPEs except for those recovered from well 3 which showed discoloration (yellow to reddish). In all cases, LDPEs were rinsed with clean water and wiped dry using clean laboratory tissue paper. Microbial activity is known to be minimal in groundwater environments (Bopp et al. 2005); this may in part account for the lack of biofilm growth on the retrieved LDPE surfaces. On all sampling occasions, the cleaned LDPEs were immediately transferred into pre-labelled cyclohexane-containing extraction vials and transported to the laboratory (on ice and in darkness) for extraction and instrumental analysis.

### Extraction and analyses of water and LDPE samples

Internal standards were added at the initiation of extraction. Water samples were extracted by horizontal shaking (100 rpm, 40 min) with cyclohexane (30 mL) while LDPE samples were extracted with hexane (30 mL) and horizontally shaken (200 rpm 72 h). Extracts were reduced by a stream of nitrogen gas to volumes appropriate for gas chromatograph (GC) and mass spectrometry (MS) (GC/MS) (i.e. ~ 1 mL).

Extracts were analysed for the target PAHs (16 U.S. EPA PAHs) and 11 deuterated PAHs on an Agilent 6890 GC equipped with a 5973 mass selective detector (Agilent Technologies, Inc., Santa Clara, CA, USA) operated in select ion monitoring mode. Analytes and internal standards were separated using an Agilent DB-5MS capillary column (30-m length, 250-μm diameter, 0.25-μm thickness, Agilent’s J&W Scientific, Santa Clara, CA, USA) and quantified with a five- or six-point calibration curve. The oven temperature was set at 40 °C for 3 min, and then elevated to 280 °C at the rate of 10 °C min^−1^ (held for 6 min). The samples were injected into the GC in splitless mode. The temperatures of the injector, electron ionisation source and MS detector were set to 250, 230 and 150 °C, respectively.

### Data analysis

The aqueous concentration of the target PAHs was estimated using the model (Eq. 1) from (Huckins et al. 2006; Smedes and Booij 2012):

1 $${C}_{w} = \frac{{N}_{PE}}{{m}_{p}*{K}_{PE-W}*DEQ}$$

where *C*_*w*_ is in units of μg L^−1^, *N*_*PE*_ is the target PAH amounts accumulated in the LDPE (μg) and *m*_*p*_ represents LDPE mass (g) while *K*_*PE-W*_ and *DEQ* are the PE-water partition coefficient (L kg^−1^) and the degree of equilibrium (unitless) reached during exposure (based on the PRCs), respectively. The *DEQ* term is used to account for non-equilibrium condition and was derived for all target PAHs using the mass transfer model (Eq. 2):

2 $$DEQ = 1-\text{exp}\left(\frac{{R}_{s}*t}{{m}_{p}*{K}_{PE-W}}\right)$$

where *R*_*s*_ is the (equivalent) water sampling rate and *t* represents exposure period (day).

The *K*_*PE-W*_ values used here were estimated from an empirical relationship derived from our previous study (in preparation, Table S3). *R*_*s*_ values were estimated using a mechanistic model (Eq. 3) derived by Rusina et al. (2010):

3 $${R}_{s}=\frac{B}{{MW}^{0.47}}$$

where *B* is an exposure-specific term that accounts for the effect of flow on uptake rate while *MW* is the molecular weight of the target analyte. The factor *MW*^0.47^ accounts for the effect of molecular size on *R*_*s*_. *B* is derived using the nonlinear least-squares (NLS) method (Booij and Smedes 2010) by fitting the PRC fraction retained (*f*_ret_) (see Target Contaminant and PRC Temporal Behaviour section) as a function of *K*_PE-W_ *M*^0.47^.

Data processing, graphing and calculations were conducted using OriginPro 2021b (OriginLab Corporation USA).

### Quality control

All samples were measured in three analytical replicates, and the resulting data are presented as the mean ± one standard deviation (unless otherwise described). Laboratory and field blank LDPE samples (*n* = 3 each) and field blank water samples (*n* = 3) were prepared and treated in a similar manner to LDPEs exposed to the wells and collected groundwater samples. No PAHs were detected above the limit of detection (LOD) in any of the blanks. Instrument LOD and quantitation (LOQ) were determined according to the German standard method DIN 32645 (Molt and Telgheder 2010). For this purpose, a calibration of 6–10 points with several points close to expected limits was performed. Recovery of target contaminants was verified by adding a defined quantity of an independent PAH mix (1000 ng) to clean water which was then treated analogously to the ground water samples. Recovery from the PEs was checked in the same way. The recovery rates were calculated by comparing determined concentrations in the extract with those of the spiked concentrations.

## Results and discussion

### TOC and DOC concentrations

TOC content in the unfiltered water samples ranged from 3 to 12 mg L^−1^ and DOC from 2.7 to 11 mg L^−1^ (Table S1).

Between 1 and 7% of the TOC was retained by the 0.45-µm filter (Table S1). These retained fractions were considered the particulate organic carbon (POC) content while the organic carbon fractions that passed through the filters (> 90%) were operationally defined as the DOC. The results indicate the studied groundwater aquifer contained very limited amounts of POC.

### Background groundwater concentrations of total PAHs based on conventional monitoring

**Fig. 1:**
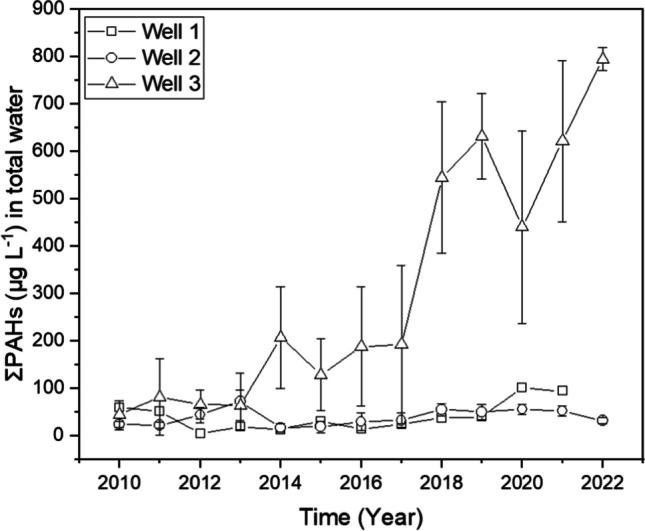
Temporal variability of ΣPAHs concentrations in total (i.e. unfiltered) water samples from groundwater wells 1, 2 and 3 based on conventional measurements from 2010 to 2022 (LMBV 2022). ΣPAHs are equivalent to the sum of 16 U.S. EPA PAHs

Long term (2010–2022) total (i.e. unfiltered) groundwater concentrations of PAHs at the three investigated groundwater wells is available. These data (Table S4; Fig. 1) were not specifically generated for this study but from a separate groundwater monitoring program (LMBV 2022). In this monitoring program, every year, each groundwater well was conventionally sampled seasonally (e.g. March, June, September, December). The resulting annual monitoring data is aimed at assessing regulatory compliance and monitoring contamination trend. Water sampling and processing were highly controlled following standard operating procedures while chemical analysis was performed by Eurofins Umwelt GmbH following German standard method (DIN 38407–39: 2011–09). These data are presented to provide readers background knowledge on the long-term total water concentrations of PAHs based on conventional measurements at the three groundwater wells and to provide a point of the comparison of contamination levels to those reported below based on equilibrium passive sampling.

The sum of PAHs (ΣPAHs) in total groundwater samples over the reporting period (2010–2022) shows a range between 4.23 and 795 μg L^−1^ (Table S4; Fig. 1). The ranking of the wells in order of contamination gives well 3 > well 1 > well 2. The variability in the yearly ΣPAHs within each groundwater well, quantified using RSD, was 77%, 65% and 381% for wells 1, 2 and 3, respectively. This suggests more constant contamination levels at wells 1 and 2 over time while levels in well 3 are increasing over time. For example, between 2010 and 2022, the ΣPAH concentrations in well 3 increased by about a factor of 8. With respect to individual PAHs, low molecular–weight (LMW) PAHs (2- and 3-ringed members) are more prevalent, three of which tend to predominate (Tables S4, S5 and S6). These, in decreasing order, were acenaphthene, fluorene and acenaphthylene. For further characterisation of individual PAHs, concentration curves of selected PAHs are presented in the supplemental data (Figure S2). Over the monitoring period (2010 to 2022), PAHs with MW no greater than that of benzo(a)anthracene (228) were consistently detected in wells 2 and 3 while other PAHs were below LOD (Tables S4, S5 and S6).

### Groundwater measured PAH total and freely dissolved concentrations

In this study, we compared the freely dissolved PAH concentrations (*C*_*w*_) measured using LDPE passive samplers with the total PAH concentrations (*C*_total_) obtained from routine monitoring. *C*_*w*_ represents a surrogate for the bioavailable fraction of PAHs in groundwater, which can be directly taken up by organisms and is more indicative of potential ecological and human health risks. In contrast, *C*_total_ includes both the freely dissolved and the particulate-bound fractions of PAHs, providing a comprehensive measure of the overall PAH burden in the environment but is not an accurate estimate of the bioavailable concentrations.

Total dissolved concentrations (*C*_total_) (i.e. DOC-bound + freely dissolved of ΣPAHs) determined from filtered water samples collected 3 days prior to deployment of LDPE passive samplers were 1010 μg L^−1^ (well 3) > 197 μg L^−1^ (well 1) > 59.7 μg L^−1^ (well 2). These values represent a range of concentration levels. *C*_total_ of individual PAHs in the filtered water samples were between 0.01 and 757 μg L^−1^ (Table 1).

**Table 1:** Total dissolved concentrations (DOC-bound + freely dissolved) in triplicate filtered water (μg L^−1^) samples and LDPE-based freely dissolved concentrations (μg L^−1^) measured at 80 days. Total dissolved samples were collected 3 days prior to deployment of passive samplers

PAHs	Well 1		Well 2		Well 3	
	*C*_total_	*C*_*w*_	*C*_total_	*C*_*w*_	*C*_total_	*C*_*w*_
Naphthalene	143.33 ± 11.55	0.05 ± 0	0.29 ± 0.14	nd	20.67 ± 2.31	0.05 ± 0.01
Acenaphthylene	19.67 ± 2.31	0.19 ± 0.14	1.01 ± 0.08	0.01 ± 0	20.67 ± 2.52	0.28 ± 0.01
Acenaphthene	4.77 ± 0.78	0.51 ± 0.58	53.33 ± 3.06	0.03 ± 0.02	756.67 ± 132.04	28.32 ± 1.08
Fluorene	4.7 ± 0.62	0.43 ± 0.31	0.45 ± 0.06	0.01 ± 0	176.67 ± 30.55	0.45 ± 0.16
Phenanthrene	6.77 ± 0.93	0.06 ± 0.02	0.08 ± 0.01	nd	20.67 ± 4.51	0.03 ± 0.01
Anthracene	1.63 ± 0.21	0.04 ± 0.02	0.26 ± 0.03	0.002 ± 0	5.1 ± 0.61	0.03 ± 0.01
Fluoranthene	8.13 ± 1.61	0.51 ± 0.11	2.87 ± 0.32	0.01 ± 0	3.63 ± 0.38	0.02 ± 0
Pyrene	4.5 ± 0.96	0.25 ± 0.04	1.37 ± 0.15	0.01 ± 0	1.47 ± 0.15	0.01 ± 0
Benzo(a)anthracene	0.97 ± 0.32	0.002 ± 0	0.01 ± 0	nd	0.01 ± 0	nd
Chrysene	0.78 ± 0.26	0.004 ± 0	0.03 ± 0.01	0.0001 ± 0	0.01 ± 0	nd
Benzo(b)fluoranthene	0.39 ± 0.16	0.0001 ± 0	nd	nd	nd	nd
Benzo(k)fluoranthene	0.38 ± 0.15	0.0001 ± 0	nd	nd	nd	nd
Benzo(a)pyrene	0.59 ± 0.25	0.0002 ± 0	nd	nd	nd	nd
Indeno(1,2,3-cd)pyrene	0.17 ± 0.09	nd	nd	nd	nd	nd
Dibenzo(a,h)anthracene	0.05 ± 0.03	nd	nd	nd	nd	nd
Benzo(ghi)perylene	0.18 ± 0.09	nd	nd	nd	nd	nd

**Fig. 2:**
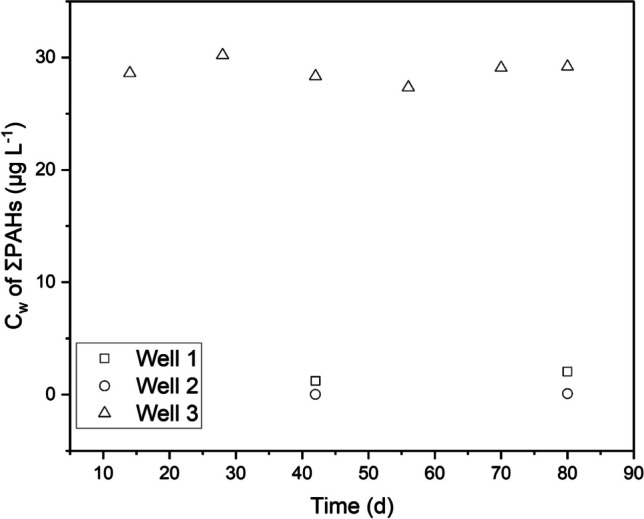
Temporal variability of freely dissolved concentrations (*C*_*w*_) of ΣPAHs measured in this study in groundwater wells 1, 2 and 3 using passive sampling. *C*_*w*_ was measured six times at well 3 but twice at wells 1 and 2. ΣPAHs are equivalent to the sum of 16 U.S. EPA PAHs

The 42-day exposure in well 1 resulted in the detection of PAHs with molecular weights no greater than that of chrysene (228) but increased to benzo(a)pyrene (252) for the 80-day exposure (Table 2). For wells 2 and 3, only PAHs with MWs up to pyrene (202) were found in quantities above the LOD for both 42- and 80-day deployments (Table 2). In general, the accumulated PAH masses were low relative to the total PAH masses present in the groundwater wells (Figs. 2 and S3). Most PAHs analysed for but not found in the LDPE (primarily the more hydrophobic PAHs) had either very low total concentrations in the ground water or were below LOD.

**Table 2:** Accumulated PAH concentrations (µg/PE) in LDPE passive samplers exposed to groundwater wells 1, 2, and 3 across two to six sampling times

PAHs	Well 1		Well 2		Well 3					
	42 day	80 day	42 day	80 day	14 day	28 day	42 day	56 day	70 day	80 day
Naphthalene	0.02 ± 0.01	0.03 ± 0	< 0,01	< 0,01	0.075 ± 0.01	0.03 ± 0.01	0.02 ± 0	0.02 ± 0.01	0.03 ± 0.01	0.03 ± 0.01
Acenaphthylene	0.24 ± 0.11	0.25 ± 0.2	0.01 ± 0	0.02 ± 0	0.503 ± 0.05	0.47 ± 0.04	0.46 ± 0.02	0.39 ± 0.08	0.38 ± 0.07	0.36 ± 0.01
Acenaphthene	0.35 ± 0.08	0.98 ± 1.12	0.03 ± 0.01	0.09 ± 0.03	54.83 ± 12.2	56.67 ± 4.16	53.33 ± 4.73	51.67 ± 7.51	55.07 ± 10.1	55.93 ± 2.09
Fluorene	0.35 ± 0.06	1.23 ± 0.89	< 0,01	0.03 ± 0.01	2.55 ± 0.75	2.00 ± 0.56	1.93 ± 0.4	1.63 ± 0.55	1.59 ± 0.8	1.51 ± 0.36
Phenanthrene	0.5 ± 0.19	1.23 ± 0.12	< 0,01	< 0,01	0.29 ± 0.11	0.32 ± 0.09	0.29 ± 0.1	0.31 ± 0.12	0.32 ± 0.14	0.29 ± 0.13
Anthracene	0.47 ± 0.21	0.73 ± 0.07	0.01 ± 0	0.02 ± 0	0.09 ± 0.02	0.16 ± 0.09	0.12 ± 0.03	0.19 ± 0.07	0.28 ± 0.08	0.29 ± 0.07
Fluoranthene	16.00 ± 0	23.22 ± 4.69	0.07 ± 0.03	0.28 ± 0.06	0.24 ± 0.11	0.43 ± 0.05	0.45 ± 0.07	0.55 ± 0.11	0.66 ± 0.09	0.77 ± 0.02
Pyrene	8.77 ± 0.38	13.03 ± 2.16	0.15 ± 0.02	0.28 ± 0.01	0.14 ± 0.05	0.23 ± 0.02	0.25 ± 0.02	0.29 ± 0.06	0.35 ± 0.04	0.40 ± 0.03
Benzo(a)anthracene	0.16 ± 0.01	0.29 ± 0.05	< 0,01	< 0,01	< 0,01	< 0,01	< 0,01	< 0,01	< 0,01	< 0,01
Chrysene	0.14 ± 0.02	0.48 ± 0.1	< 0.01	< 0.01	< 0.01	< 0.01	< 0.01	< 0.01	< 0.01	< 0.01
Benzo(b)fluoranthene	< 0.01	0.03 ± 0.01	< 0.01	< 0.01	< 0.01	< 0.01	< 0.01	< 0.01	< 0.01	< 0.01
Benzo(k)fluoranthene	< 0.01	0.02 ± 0	< 0.01	< 0.01	< 0.01	< 0.01	< 0.01	< 0.01	< 0.01	< 0.01
Benzo(a)pyrene	< 0.01	0.05 ± 0.02	< 0.01	< 0.01	< 0.01	< 0.01	< 0.01	< 0.01	< 0.01	< 0.01
Indeno(1,2,3-cd)pyrene	< 0.01	< 0.01	< 0.01	< 0.01	< 0.01	< 0.01	< 0.01	< 0.01	< 0.01	< 0.01
Dibenzo(a,h)anthracene	< 0.01	< 0.01	< 0.01	< 0.01	< 0.01	< 0.01	< 0.01	< 0.01	< 0.01	< 0.01
Benzo(ghi)perylene	< 0.01	< 0.01	< 0.01	< 0.01	< 0.01	< 0.01	< 0.01	< 0.01	< 0.01	< 0.01

**Fig. 3:**
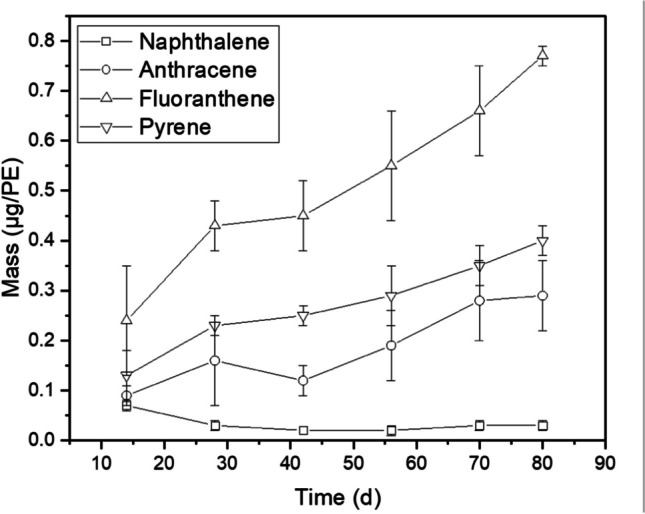
Time series of selected PAH masses accumulated in the LDPE passive samplers exposed to groundwater in well 3

Data from the extended time series passive sampling of well 3 (Table 2, Table S7) allowed further assessment of the PAH uptake dynamics. Accumulation curves for selected PAHs from the samplers exposed to well 3 over the 80-day exposure period are shown in Fig. 3.

As can be seen in Fig. 3, similar masses of naphthalene were absorbed over the 80-day exposure; this is indicative of a membrane-controlled uptake kinetics (Allan et al. 2010). In contrast, the accumulation of anthracene, fluoranthene and pyrene tended to follow a gradual increasing trend, which suggests a linear uptake and that most of the mass transfer resistance is likely boundary-layer controlled. About 0.03 µg, 0.29 µg, 0.40 µg and 0.77 µg of naphthalene, anthracene, pyrene and fluoranthene, respectively, were found in the LDPE exposed to well 3 for 80 days. The faster accumulation of fluoranthene relative to the other PAHs (Fig. 3) may be due to differences in the sampling rate (*R*_*s*_) and/or variations in the dissolved concentrations of these PAHs.

It is important to emphasize that the accumulated levels of certain monitored PAHs, particularly acenaphthylene and fluorene, consistently exhibit a decline over time, specifically in well 3. This trend sharply contrasts with the behaviour of other PAHs and the data recorded in wells 1 and 2. The distinctive pattern observed in well 3 may, in part, be ascribed to the significant variability in the annual total concentration, as illustrated in Fig. 1. This anomaly appears exclusive to well 3 and could potentially be linked to challenges associated with a flow regime influenced by seasonal factors.

Dissipation of PRCs from the LDPE exposed to the three wells was observed, allowing the derivation and assessment of the fractions of retained PRCs, *f*_ret_:

4 $${f}_{\text{ret}} = \left(\frac{{N}_{t}}{{N}_{0}}\right)=\text{exp}\left(\frac{B*t}{{m}_{p}*{K}_{PE-W}*{M}^{0.47}}\right)$$

where *N*_*t*_ and *N*_0_ are the PRC amounts in the PEs prior and after the deployment.

**Fig. 4:**
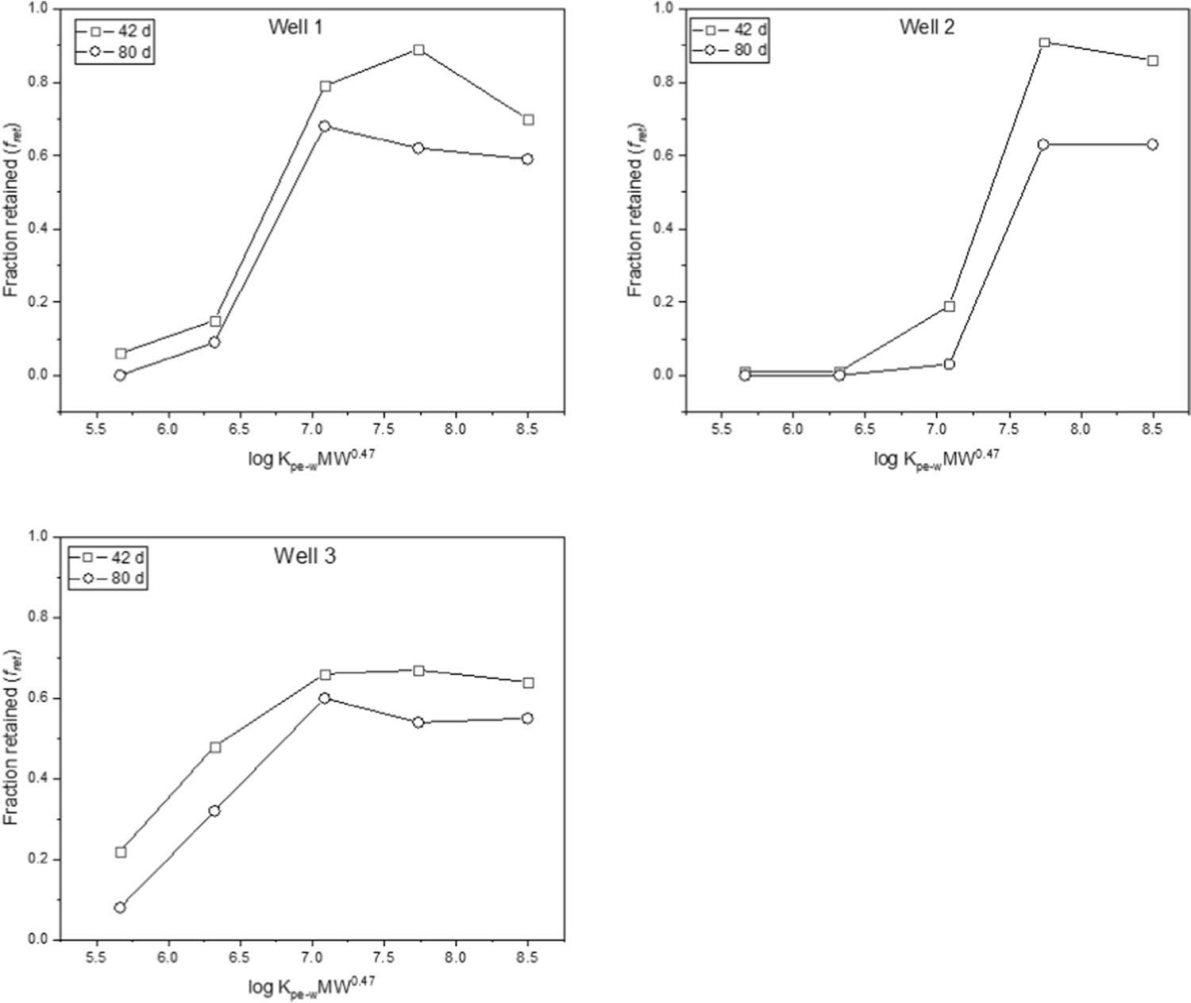
Fraction of retained PRCs as a function of log *K*_PE-W_ *MW*.^0.47^ for groundwater wells 1, 2 and 3 at days 42 and 80

Using Eq. 3, the estimated *f*_ret_ values range from 0 to 1 depending on the PRC and the exposure time point (Table S8). To assess the *f*_ret_ trends between the three exposure wells, *f*_ret_ values for 42 days and 80 days were plotted as a function of log *K*_PE-w_ *MW*^0.47^ (Fig. 4).

From Fig. 4, it can be observed the* f*_ret_ curves differ between the exposure wells. The PRCs *f*_ret_ at 42 days were higher compared to those at 80-day exposures thus suggesting the *f*_ret_ decreased with time. Also, the *f*_ret_ values tend to increase with increasing *K*_PE-W_ for all the PRCs except the two largest PRCs (i.e. d12-benzo(a)pyrene and d14-dibenzo(a,h)anthracene). In principle, a linear relationship is expected between the retained fractions of the PRCs and log *K*_PE-W_ *M*^0.47^ not an inverse correlation as exhibited by the two largest PRCs (Fig. 4). This inverse correlation is unusual and was observed at all exposure wells and time points (Figure S4). A direct inference is that the *f*_ret_ values of the two largest PRCs are lower than expected (i.e. their dissipated amounts are higher than expected). This is more evident when the *f*_ret_ values of the two largest PRCs are compared with those of d12-benzo(a)anthracene (a PRC with *K*_PE-W_ more than an order of magnitude less than those of the two largest PRCs).The source of this unusual relationship is not completely known but may be connected to experimental artefacts (e.g. matrix-effect, incomplete extraction) (Muz et al. 2021; Perron et al. 2013).

Further assessment of the *f*_ret_ − log *K*_PE-W_ *M*^0.47^ relationship was conducted. This involved fitting of the modelled *f*_ret_ to measured data using Eq. 3. A graphical representation of the results of this assessment is presented in Figure S5. As shown in Figure S5, the model fit the measured *f*_ret_ of all the PRCs except the two largest PRCs compared fairly well and was characterised with residual errors (i.e. difference between measured and modelled *f*_ret_) which are centred randomly around zero (Figure S6) except for the two larger PRCs characterised with residual errors that are not random but inversely related to *K*_PE-W_.

### Estimation of the degree of equilibrium, sampling rate and mass transfer kinetics

As shown in Eq. 1, the DEQ attained during exposure is critical to the precise estimation of *C*_*w*_ so that it is not over or understated. The mass transfer model (Eq. 2), calibrated by the PRC data, was used to estimate the DEQ attained for all target PAHs during the deployments. The DEQ data (Table S9) shows that equilibrium conditions were reached, based on DEQ > 0.9 (Joyce and Burgess 2018) after the 42-day exposure for PAHs with MW no greater than anthracene (178) for groundwater wells 1 and 3 and chrysene (228) for groundwater well 2. This finding suggests PE-groundwater equilibrium conditions are attained more rapidly at wells 2 than at wells 1 and 3. The DEQ values show, as expected, a decrease with increasing PAH molecular size indicating that high molecular–weight (HMW) PAHs require longer time to equilibrate relative to their LMW counterparts. Also, the DEQ results further highlight the importance of using PRCs of varying molecular size to account for equilibrium conditions during exposure.

**Fig. 5:**
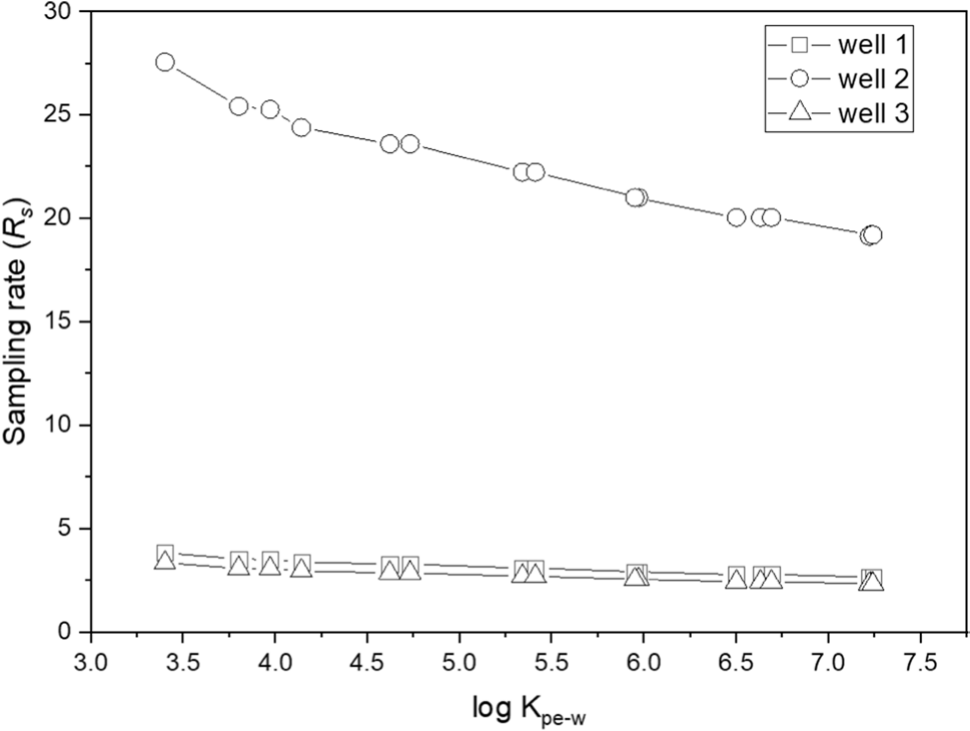
Estimated sampling rate (*R*_*s*_) after an 80-day deployment period versus log *K*_pe-w_ in groundwater wells 1, 2 and 3

The *R*_*s*_ for the target PAHs at all three groundwater wells were estimated and ranged from 2.34–27.58 Ld^−1^ (Fig. 5). In general, the *R*_*s*_ values decreased with increasing log *K*_PE-W_. The *R*_*s*_ curves of groundwater wells 1 and 3 are characterised with a gentle slope while that of well 2 has a steep slope (Fig. 5).

The mean *R*_*s*_ at well 2 (22.15 Ld^−1^) is seven and eight times more than those encountered at wells 1 and 3, respectively. The higher *R*_*s*_ values at well 2 may, in part, explain the faster equilibrium attainment and the higher PRC dissipation (i.e. lower values of PRC *f*_ret_) encountered in this well relative to wells 1 and 3. These *R*_*s*_ values likely indicate the energy levels (i.e. turbulence intensity, direction of flow relative to the LDPE surface area) at each exposure well, with well 2 being the most dynamic (higher energy) followed by the less energetic wells 1 and 3.

To characterise the mass transfer processes operating under the conditions at the three exposure groundwater wells, we considered PAH accumulation into the LDPE as a multi-transport process with the resistance to overall mass transfer (1/*k*_o_) represented as follows:

5 $$\frac{1}{{k}_{0}}=\frac{1}{{k}_{w}}+\frac{1}{{{k}_{w}K}_{PE-W}}$$

where *k*_w_ and *k*_m_ are the mass transfer coefficients of the water boundary layer (WBL) and the LDPE membrane, respectively (Booij and Smedes 2010).

A comparison of the relative importance of mass transport through the LDPE membrane (*I*_PE_) and the WBL (*I*_*w*_) can be developed based on Booij et al. (2017):

6 $$\frac{{I}_{\text{PE}}}{{I}_{w}}=\frac{{k}_{w}*L}{{D}_{\text{PE}}*{K}_{\text{PE}-\text{W}}*\rho }$$

where *D*_PE_ is the diffusion coefficient in the LDPE, *ρ* (0.91 kg L^−1^) is the density of the polymer, and *L* (35 μm) is the half-thickness of the PE. *D*_PE_ was estimated using the relational equation between *D*_PE_ and the McGowan molar volume of the target PAHs (Booij et al. 2017):

7 $${\text{log}D}_{\text{PE}}=0.0145 {V}_{\text{McGowan}}-10.43$$

Given *K*_*w*_ can be considered as a surface area normalised sampling rate, the model (Eq. 8) was used to derive the *k*_*w*_ values:

8 $${k}_{w}={~}^{B}\!\left/ \!{~}_{A{MW}^{0.47}}\right.$$

where *A* (0.66 dm^2^) is the sampler area exposed to the water during the deployment. The determination of *k*_*w*_ allowed the estimation of WBL thickness (*δ*_*w*_):

9 $${\updelta }_{w}={~}^{{D}_{w}}\!\left/ \!{~}_{{k}_{w}}\right.$$

where *D*_*w*_ is the diffusion coefficient of the target contaminant in water. *D*_*w*_ values are estimated based on the following equation using the contaminant’s molecular weight (MW) (g mol^−1^) (Lohmann 2012):

10 $${\text{log}D}_{w}=-7.57-0.71log\;MW$$

Equation 8 predicts *k*_w_ values between 3.54 and 41.76 µm s^−1^ (Table S10) depending on the PAH and exposure well. Exposure well 3 had the lowest *k*_w_ values, followed by well 1, and the highest are encountered at well 2. Given that the magnitude of *k*_w_ is dependent on the effects of hydrodynamic flow, sampler design and target contaminant properties (Eq. 6), it is difficult to compare *k*_w_ reported in other studies. However, to give a perspective, a similar range of *k*_w_ values (2- 28 µm s^−1^) has been previously reported for field-exposed LDPE samplers (Estoppey et al. 2014; McDonough et al. 2016; Vrana et al. 2007; Joyce and Burgess 2018).

Results from the application of Eq. 6 show that the *I*_*PE*_*/I*_*W*_ values ranged from 0.0002 to 0.645 (Table S10). Based on the criteria that fully membrane-controlled exchange can be expected when *I*_*PE*_*/I*_*W*_ >  > 1 (Booij et al. 2017), it can be inferred from the empirical *I*_*PE*_*/I*_*W*_ data that the transition to fully WBL-controlled exchange kinetic occurred at acenaphthene for groundwater wells 1 and 3 but shifted to fluoranthene for well 2. The point at which the transition to complete WBL control occurs for well 2 is largely due to higher *k*_w_ values encountered in this well (Vrana and Schüürmann 2002) compared to wells 1 and 3. Here, the result to of the PRC-based assessment (i.e. *I*_*PE*_*/I*_*W*_ data) of the phase (i.e., WBL or polymer) influencing and controlling PAH exchange kinetics between the LDPE and groundwater is in agreement with that investigated through PAH masses absorbed by the LDPE.

From Eq. 9, estimates of the *δ*_*w*_ ranged from 17 to 168 µm (Table S10) and are in agreement with those previously reported or estimated (Joyce and Burgess 2018; Apell et al. 2016; Lohmann 2012). The *δ*_*w*_ values encountered at wells 1 and 3 are in line with those expected of a laminar flow regime while those of well 2 are typical of a turbulent flow regime (Vrana and Schüürmann 2002).

### Groundwater flow effects on polyethylene sampling kinetics

A high sampling rate (*R*_*s*_) is often essential for good passive sampler performance; however, in cases where *R*_*s*_ exceeds the ambient water flow rate, this may result in rapid reduction of contaminants in the area surrounding the passive sampler due to depletive sampling. This phenomena is particularly possible under quiescent flow conditions (e.g. in groundwater with low aquifer permeability and/or a low filter zone). During passive sampling in the field, a direct effect of depletive sampling may include reduced in situ *R*_*s*_ relative to the expected laboratory-derived *R*_*s*_ values (Vrana et al. 2005b).

To determine if the current study involved depletive sampling, we compared the actual in situ *R*_*s*_ data and the groundwater flow rate, *Q*. To determine a value for *Q*, the following equation was applied:

11 $$Q=Ki{A}_{e}$$

*K* is the hydraulic conductivity and *i* stands for the hydraulic gradient while *A*_*e*_ is cross-sectional area calculated as the depth of the groundwater well times twice its radius (Gustavson and Harkin 2000). The *K* and *i* values were available from previous site assessments and not specifically obtained for this study.

As earlier mentioned, the estimated in situ *R*_*s*_ values were between 2.34 and 27.58 Ld^−1^ while the calculated *Q* values ranged from 144 to 348 Ld^−1^. The *R*_*s*_ values represent the volume of water. and the LDPE has the potential to clear per day. These *R*_*s*_ values are much lower than the daily turnover rates encountered in the groundwater wells examined in this study. The residence time, *t*_*r*_ (h), of water in the sampling wells, estimated as the ratio of groundwater reservoir (i.e. volume) to the flux (i.e., groundwater flow rate), was between 3.1 and 4.2 h. This means the groundwater wells are completely refreshed every 3 to 4 h; under this condition, the aqueous concentration of the target contaminant likely remained constant during the LDPE deployment period. From this analysis, it can be concluded that the LDPE sampling kinetics were not limited by groundwater flow, and depletive sampling was unlikely to have occurred.

### Comparison of measured freely dissolved concentrations with regulatory standards

In this section, we provide a comparison of our *C*_*w*_ data (Table 1; Fig. 2) with the German regulatory benchmark known as the “Insignificant Threshold Concentration” (German: Geringfügigkeitschwelle, GFS) as outlined in the LAWA (German Working Group on Water Issues) guidelines of 2016. The GFS value, established at 0.2 μg L^−1^ for ΣPAHs (without naphthalene), in groundwater signifies a concentration threshold below which the presence of a substance is deemed inconsequential to groundwater chemical quality and poses no discernible risks to human health or the environment (LAWA 2016). The results of our ΣPAHs *C*_*w*_ data show that groundwater well 2 conspicuously adheres to the GFS value, effectively reflecting a concentration below which regulatory concerns are minimal. In stark contrast, groundwater wells 1 and 3 exhibit ΣPAHs *C*_*w*_ surpassing the GFS value by 10 and 145 times, respectively. From an eco- and human toxicity perspective, this means wells 1 and 3 are highly unfit for human consumption and are indicative of deleterious exposure to groundwater dwelling organisms.

## Conclusions

An evaluation of equilibrium passive sampling in three contaminated groundwater wells showed successful uptake of the target PAHs over a wide range of concentrations. The estimated *C*_*w*_ ranged from 0.07 to 29.2 μg L^−1^ for ΣPAHs which is substantially less than the *C*_total_ which ranged from 59.7 to 1010 μg L^−1^ ΣPAHs. The *C*_total_ also reflected ΣPAH concentrations reported by routine groundwater monitoring performed with conventional methods. By comparing these two concentrations, we gain insights into the distribution of PAHs between freely dissolved and other aqueous phases, which is important for risk assessment and remediation strategies. This comparison highlights the complementary nature of using both passive samplers and routine monitoring to obtain a fuller picture of PAH contamination in groundwater.

The evaluation of *C*_*w*_ data against the GFS regulatory standard highlights a significant difference between what the regulations specify and what our actual measurements reveal. The alignment of well 2 with the GFS standard reaffirms its compliance with groundwater quality benchmarks. However, the pronounced deviation of wells 1 and 3 from the GFS standard underscores their unsuitability for human consumption and highlights ecological vulnerability. This emphasizes the need for continued monitoring and management to maintain the investigated area’s groundwater integrity, protect human health, and preserve ecological balance.

The estimates of exchange rate parameters, derived from the PRC dissipation data, are similar for PAHs of similar size and hydrophobicity. These values are very similar for groundwater wells 1 and 3 but differed from those of groundwater well 2 by a factor of 8. The PRC data confirmed the transition from membrane- to boundary layer–controlled exchange occurred at acenaphthene for groundwater wells 1 and 3 but fluoranthene for groundwater well 2. This observation is consistent with the exchange kinetics assessed based on the masses of accumulated PAHs in the LDPE. The daily turnover volume encountered in the groundwater wells was 7 to 1000 times more than the estimated in situ LDPE sampling rates for the target contaminants ruling-out depletive sampling during the deployments.

To date, few studies have been performed evaluating the use of passive sampling in groundwater, and this study improves and advances our understanding of equilibrium passive sampling in the monitoring of PAHs in groundwater and demonstrates its suitability for future use in monitoring groundwater contamination. However, the use of LDPE with thinner thicknesses (e.g. 30 μm) is recommended. This would help shorten the equilibration time, since diffusion in the LDPE along with its thickness is the primary drivers of equilibration.

## Supplementary Information

Below is the link to the electronic supplementary material.

Supplementary file1 (DOCX 1124 KB)

## Data Availability

All data supporting the findings of this study in its current form are available within the paper and supplied supplementary materials.
