# Supplementary material for: Application of low-density polyethylene (LDPE) passive samplers for monitoring PAHs in groundwater
Source: Environ Sci Pollut Res Int. 2024 Aug 29;31(42):54546–58. doi: 10.1007/s11356-024-34731-7 (PMC11413203; doi:10.1007/s11356-024-34731-7)
Supplement: Supplementary file 1 — Supplementary file1 (DOCX 1124 KB) [file 11356_2024_34731_MOESM1_ESM.docx]

**Application of Low Density Polyethylene (LDPE) Passive Samplers for Monitoring PAHs in Groundwater**

**Ibukun Olaa***†**, Carsten Drebenstedta, Robert M. Burgess^b^ , Ian J. Allan^c^, Nils Hotha, and Christoph Küllsc**

*aInstitute of Mining and Special Civil Engineering, Technical University Mining Academy, Gustav-Zeuner Street 1A, Freiberg, 09599, Germany*

*^b^U.S.* *Environmental Protection Agency, Office of Research and Development, Center for Environmental Measurement and Modeling, Atlantic Coastal Environmental Sciences Division, 27 Tarzwell Drive, Narragansett, Rhode Island 02882, United States*

*cNorwegian Institute for Water Research (NIVA),* *Økernveien 94, Oslo NO-0579, Norway*

*dLabor für Hydrologie und Internationale Wasserwirtschaft, Technische Hochschule*

*Lübeck, Lübeck, 23562, Schleswig-Holstein, Germany*

Corresponding author.

[Ibukun.Ola@doktorand.tu-freiberg.de](mailto:Ibukun.Ola@doktorand.tu-freiberg.de)

**Supplementary Material**

**Table S1**. Sampling wells and selected geochemical and geophysical features including measured total organic carbon (TOC) and dissolved organic carbon (DOC).

| Sampling well | Well depth (m) | Water depth (m) | Screen interval (m) | Hydraulic gradient (unitless) | Hydraulic conductivity  (m s^-1^) | TOC  (mg L^-1^ ) | DOC  (mg L^-1^ ) | POC  (mg L^-1^ ) |
| --- | --- | --- | --- | --- | --- | --- | --- | --- |
| Well 1 | 9.0 | 3.69 | 5.2 – 8.1 | 0.0143 | 6.10E-04 | 12.4±0.21 | 11.5±0.06 | 0.9 |
| Well 2 | 7.2 | 4.58 | 3.0 – 6.0 | 0.0143 | 4.50E-04 | 3.00±0.14 | 2.97±0.06 | 0.03 |
| Well 3 | 4.5 | 1.60 | 1.9 – 3.9 | 0.0185 | 4.50E-04 | 5.13±0.21 | 4.97±0.12 | 0.16 |

**Table S2.** Initial concentrations of PRCs in replicate LDPE passive samplers.

| Chemical | | µg PE^-1^ |  |  | | Average ± SD |
| --- | --- | --- | --- | --- | --- | --- |
|  | Replicate Sampler | | | |  | |
|  | | 1 | 2 | 3 | |  |
| Anthracene-D10 | | 1.7 | 1.6 | 1.6 | | 1.63±0.06 |
| Fluoranthene-D10 | | 2.8 | 2.5 | 2.61 | | 2.64±0.15 |
| Benzo(a)anthracene-D12 | | 1.59 | 1.29 | 1.07 | | 1.32±0.26 |
| Benzo(a)pyrene-D12 | | 2.05 | 2.31 | 1.91 | | 2.09±0.2 |
| Dibenzo(a,h)anthracene-D14 | | 2.08 | 2.61 | 2.38 | | 2.36±0.27 |

**Table S3.** Physicochemical properties of the target analyte.

| PAHs | Abbreviation | log K_ow_^a,b^  Lkg^-1^ | MW  g mol^–1^ | log K_PE-W_^c^  Lkg^-1^ | D_PE_^d^  µm^2^/s | D_W_^e^  µm^2^/s | V_M_^f^  cm^3^ mol^-1^ |
| --- | --- | --- | --- | --- | --- | --- | --- |
| Naphthalene | Nap | 3.40 | 128.20 | 3.01 | 0.99 | 857.83 | 108.54 |
| Acenaphthylene | Acy | 3.80 | 152.20 | 3.41 | 0.64 | 759.43 | 121.56 |
| Acenaphthene | Ace | 3.97 | 154.20 | 3.58 | 0.56 | 752.42 | 125.86 |
| Fluorene | Flu | 4.14 | 166.20 | 3.74 | 0.40 | 713.43 | 135.65 |
| Phenanthrene | Phen | 4.62 | 178.20 | 4.22 | 0.29 | 678.98 | 145.44 |
| Anthracene | Ant | 4.73 | 178.20 | 4.33 | 0.29 | 678.98 | 145.44 |
| Fluoranthene | Flt | 5.34 | 202.30 | 4.93 | 0.19 | 620.50 | 158.46 |
| Pyrene | Pyr | 5.41 | 202.30 | 5.00 | 0.19 | 620.50 | 158.46 |
| Benzo(a)anthracene | B(a)A | 5.97 | 228.30 | 5.56 | 0.08 | 569.46 | 182.34 |
| Chrysene | Cry | 5.95 | 228.30 | 5.54 | 0.08 | 569.46 | 182.34 |
| Benzo(b)fluoranthene | B (b)F | 6.50 | 252.30 | 6.08 | 0.05 | 530.44 | 195.36 |
| Benzo(k)fluoranthene | B(k)F | 6.63 | 252.30 | 6.21 | 0.05 | 530.44 | 195.36 |
| Benzo(a)pyrene | B(a)p | 6.69 | 252.30 | 6.27 | 0.05 | 530.44 | 195.36 |
| Indeno(1,2,3-cd)pyrene | IP | 7.23 | 276.30 | 6.80 | 0.04 | 497.30 | 208.38 |
| Dibenzo(a,h)anthracene | D (a,h)A | 7.22 | 278.30 | 6.79 | 0.02 | 494.76 | 219.24 |
| Benzo(ghi)perylene | B (ghi)P | 7.24 | 276.30 | 6.81 | 0.04 | 497.30 | 208.38 |

a (Ma et al., 2010).

b (Jonker, 2016).

c K_PE-W_ values calculated through an empirical relationship (0.9901log K_ow_ - 0.355) derived from our previous study (in preparation).

d Polyethylene diffusivity values calculated from: log(D_PE_) = 0.0145 V_Mc Gowan_ - 10.43 (Booij et al., 2017).

e Water diffusivity (D_W_) values calculated from : log(D_w_) = -7.57 – 0.71log MW (Lohmann, 2012).

f Molar volume (V_M_) (Abraham and McGowan, 1987).


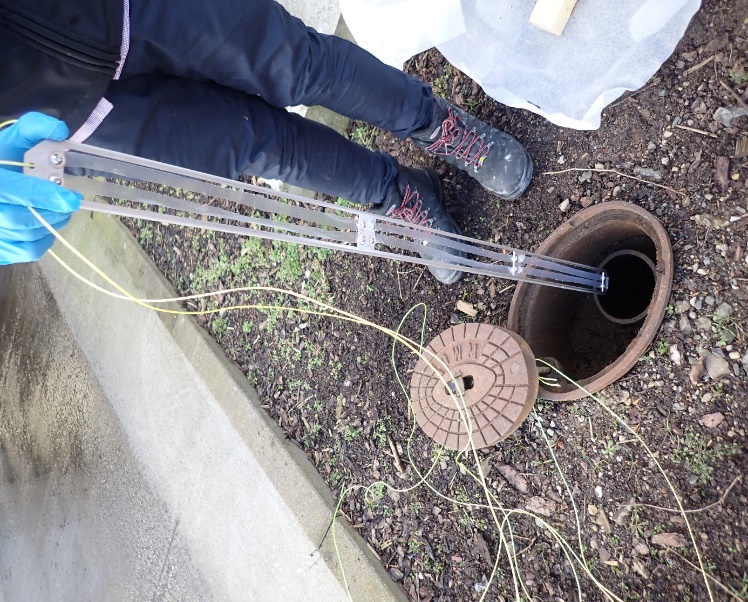

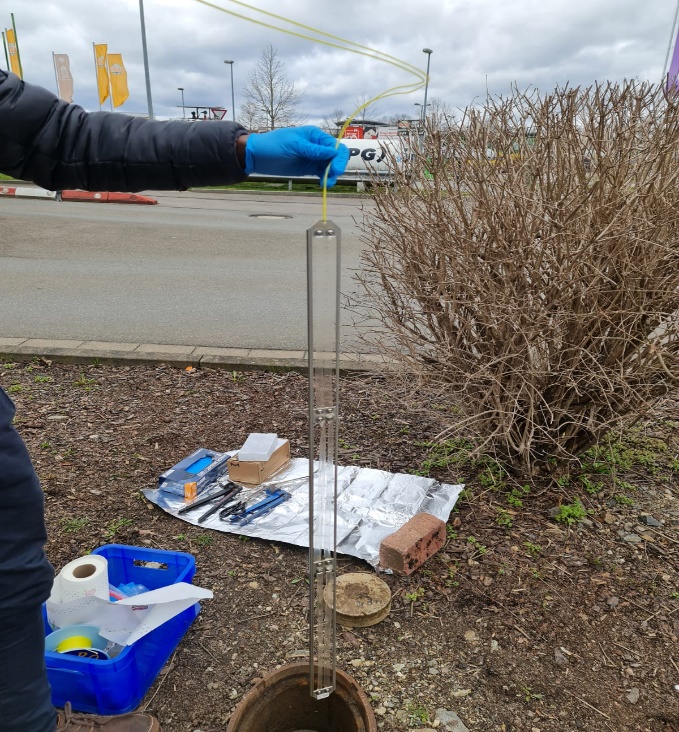


Well 2

Well 1


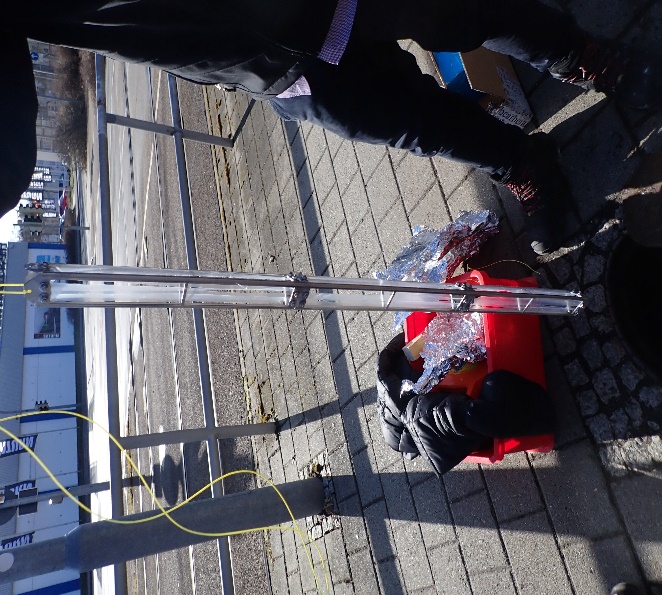


Well 3

**Figure S1.** Image of LDPE deployment systems for groundwater in wells 1, 2 and 3.

**Table S4.** Summary statistics of ΣPAHs concentrations (μg L^-1^) in total water samples (2010 – 2022) in groundwater wells 1, 2 and 3 based on conventional measurement (LMBV, 2022).

|  | Well 1 | | | Well 2 | | | Well 3 | | |
| --- | --- | --- | --- | --- | --- | --- | --- | --- | --- |
| Year | Minimum | Maximum | Average ± SD | Minimum | Maximum | Average ± SD | Minimum | Maximum | Average ± SD |
| 2010 | NA | NA | 58.45 | 14.93 | 28.69 | 23.1±5.91 | 1.95 | 75.2 | 43.54±30.5 |
| 2011 | NA | NA | 50.4225 | 13.42 | 25.88 | 21.91±5.74 | 7.97 | 183.39 | 81.39±80.17 |
| 2012 | NA | NA | 4.235 | 19.74 | 54.61 | 43.63±16.08 | 39.06 | 110.29 | 65.75±31.14 |
| 2013 | NA | NA | 18.7075 | 14.61 | 14.,44 | 72.06±59.65 | 31 | 97.06 | 63.79±32.05 |
| 2014 | NA | NA | 12.88 | 8.85 | 26.59 | 16.3±8.2 | 17.59 | 663.36 | 206.99±307.33 |
| 2015 | NA | NA | 30.625 | 3.11 | 29.18 | 18.49±12.02 | 45.99 | 225.55 | 128.42±75.06 |
| 2016 | NA | NA | 13.335 | 1.89 | 45.04 | 29.08±19.08 | 3.54 | 284.34 | 187.98±125.89 |
| 2017 | NA | NA | 24.145 | 8.9 | 42.88 | 31.83±15.54 | 51.15 | 428.92 | 192.18±167.14 |
| 2018 | NA | NA | 36.825 | 40.9 | 71.27 | 54.6±12.64 | 419.41 | 760.18 | 544.65±159.06 |
| 2019 | NA | NA | 39.275 | 38.65 | 72.76 | 49.39±15.78 | 503.52 | 712.65 | 631.87±90.59 |
| 2020 | NA | NA | 101.4 | 40.16 | 64.4 | 55.1±10.91 | 295.43 | 741.59 | 440.26±203.52 |
| 2021 | NA | NA | 93.75 | 39.21 | 62.91 | 51.56±9.93 | 377.11 | 763.5 | 621.77±170.05 |
| 2022^*^ | NA | NA | NA | 27.09 | 36.17 | 31.63±6.42 | 777.9 | 811.45 | 794.68±23.72 |

NA, not available

**Table S5.** Concentrations of individual PAHs (μg L^-1^) in total water samples in groundwater well 2 based on conventional measurement (LMBV, 2022).

| Compound | 2010 | 2011 | 2012 | 2013 | 2014 | 2015 | 2016 | 2017 | 2018 | 2019 | 2020 | 2021 | 2022^*^ |  |
| --- | --- | --- | --- | --- | --- | --- | --- | --- | --- | --- | --- | --- | --- | --- |
| Nap | 0.43±0.31 | 0.38±0.42 | 0.31±0.27 | 13.17±25.89 | 0.48±0.43 | 0.31±0.23 | 0.18±0.05 | 0.17±0.14 | 0.31±0.18 | 0.45±0.48 | 1.63±0.67 | 0.47±0.3 | 0.07±0.01 |  |
| Acy | 0.66±0.3 | 0.44±0.12 | 0.81±0.28 | 0.93±0.81 | 0.29±0.16 | 0.33±0.24 | 0.45±0.28 | 0.67±0.32 | 0.95±0.31 | 0.99±0.21 | 1.01±0.25 | 0.89±0.18 | 0.83±0.21 |  |
| Ace | 20±6.06 | 19.25±4.99 | 39±14.09 | 49±48.37 | 13.3±6.8 | 15.43±10.76 | 25.35±16.71 | 26.53±13.13 | 48.5±11.27 | 42.75±15.52 | 48±10.36 | 46±9.31 | 27.5±4.95 |  |
| Flu | 0.51±0.21 | 0.29±0.12 | 0.39±0.24 | 5.84±7.32 | 0.45±0.27 | 0.22±0.17 | 0.27±0.31 | 0.26±0.17 | 0.33±0.15 | 0.63±0.46 | 0.71±0.23 | 0.5±0.12 | 0.2±0.04 |  |
| Phen | 0.12±0.04 | 0.06±0.06 | 0.08±0.04 | 1.04±1.45 | 0.26±0.14 | 0.12±0.08 | 0.2±0.19 | 0.18±0.15 | 0.22±0.1 | 0.59±0.62 | 0.55±0.29 | 0.38±0.04 | 0.22±0.12 |  |
| Ant | 0.14±0.06 | 0.11±0.03 | 0.16±0.06 | 0.46±0.43 | 0.11±0.04 | 0.1±0.06 | 0.16±0.1 | 0.21±0.1 | 0.31±0.12 | 0.29±0.11 | 0.21±0.06 | 0.19±0.06 | 0.15±0.02 |  |
| Flt | 0.83±0.33 | 0.91±0.28 | 1.86±0.87 | 1.01±0.29 | 0.91±0.55 | 1.17±0.91 | 1.58±1.04 | 2.1±1.02 | 2.63±0.96 | 2.3±0.57 | 1.98±0.44 | 2.13±0.41 | 1.8±0.71 |  |
| Pyr | 0.59±0.37 | 0.47±0.14 | 1.01±0.49 | 0.56±0.23 | 0.49±0.31 | 0.65±0.48 | 0.81±0.54 | 1.08±0.53 | 1.32±0.49 | 1.15±0.27 | 0.97±0.22 | 0.98±0.21 | 0.84±0.37 |  |
| B(a)A | 0.02±0.01 | 0.01±0 | 0.02±0.01 | 0.04±0.03 | 0.03±0 | 0.03±0.03 | 0.07±0.08 | 0.52±0.85 | 0.03±0.01 | 0.05±0.04 | 0.02±0.01 | 0.03±0.01 | 0.02±0.01 |  |
| Cry | 0.02±0 | <0.01 | 0.03±0.01 | 0.03±0.03 | <0.01 | 0.06±0.06 | 0.03±0.02 | 0.33±0.54 | 0.02±0 | 0.04±0.04 | 0.02±0.01 | 0.02±0 | 0.02±0.01 |  |
| B (b)F | <0.01 | <0.01 | <0.01 | <0.01 | <0.01 | <0.01 | <0.01 | <0.01 | <0.01 | <0.01 | <0.01 | <0.01 | <0.01 |  |
| B(k)F | <0.01 | <0.01 | <0.01 | <0.01 | <0.01 | <0.01 | <0.01 | <0.01 | <0.01 | <0.01 | <0.01 | <0.01 | <0.01 |  |
| B(a)p | <0.01 | <0.01 | <0.01 | <0.01 | <0.01 | <0.01 | <0.01 | <0.01 | <0.01 | <0.01 | <0.01 | <0.01 | <0.01 |  |
| IP | <0.01 | <0.01 | <0.01 | <0.01 | <0.01 | <0.01 | <0.01 | <0.01 | <0.01 | <0.01 | <0.01 | <0.01 | <0.01 |  |
| D (a,h)A | <0.01 | <0.01 | <0.01 | <0.01 | <0.01 | <0.01 | <0.01 | <0.01 | <0.01 | <0.01 | <0.01 | <0.01 | <0.01 |  |
| B (ghi)P | <0.01 | <0.01 | <0.01 | <0.01 | <0.01 | <0.01 | <0.01 | <0.01 | <0.01 | <0.01 | <0.01 | <0.01 | <0.01 |  |

** data derived from two measurements (additional sampling campaigns are scheduled for August and November 2022)*

**Table S6.** Concentrations of individual PAHs (μg L^-1^) in total water samples (2010 – 2022) in groundwater well 3 based on conventional measurement (LMBV, 2022).

|  | Compound | 2010 | 2011 | 2012 | 2013 | 2014 | 2015 | 2016 | 2017 | 2018 | 2019 | 2020 | 2021 | 2022^*^ |
| --- | --- | --- | --- | --- | --- | --- | --- | --- | --- | --- | --- | --- | --- | --- |
|  | **Nap** | 0.79±0.36 | 0.63±0.53 | 0.33±0.26 | 3.9±7.4 | 0.39±0.33 | 0.79±0.47 | 0.9±1.14 | 0.97±0.73 | 1.27±1.36 | 0.53±0.52 | 0.71±0.37 | 2.33±2.95 | 18±2.83 |
|  | **Acy** | 4.46±7.71 | 0.93±0.86 | 0.76±0.24 | 0.93±0.44 | 2.16±2.84 | 2.35±1.81 | 2.83±2.06 | 4.77±3.88 | 12.5±1.29 | 9.63±0.99 | 7.33±2.68 | 13.35±6.73 | 27±2.83 |
|  | **Ace** | 30.85±21.3 | 70.5±70.48 | 57.5±28.21 | 51±22.55 | 180.25±269.17 | 109±63.42 | 160.01±109.82 | 157.75±147.24 | 462.5±141.51 | 557.5±97.43 | 375±193.65 | 550±198.49 | 575±49.5 |
|  | **Flu** | 6.24±4.55 | 8.19±8.04 | 6.35±2.73 | 6.02±5.17 | 22±32.28 | 14.88±8.93 | 20.75±14.68 | 25.2±14.94 | 59.25±18.55 | 57±10.46 | 51±10.42 | 48.5±17.82 | 150±14.14 |
|  | **Phen** | 0.38±0.17 | 0.4±0.31 | 0.33±0.12 | 0.6±0.81 | 0.4±0.38 | 0.29±0.15 | 0.77±0.66 | 1±0.64 | 1.63±1.14 | 0.88±0.29 | 0.72±0.24 | 1.74±1.62 | 16.5±4.95 |
|  | **Ant** | 0.29±0.25 | 0.34±0.29 | 0.23±0.15 | 0.28±0.12 | 0.75±0.93 | 0.5±0.28 | 0.94±0.62 | 1.07±0.85 | 2.58±0.38 | 2.13±0.42 | 1.5±0.34 | 1.43±0.85 | 4.2±0.85 |
|  | **Flt** | 0.26±0.21 | 0.27±0.24 | 0.17±0.09 | 0.69±0.88 | 0.73±1 | 0.43±0.33 | 0.87±0.12 | 0.99±0.87 | 3.4±0.85 | 2.95±0.81 | 2.83±0.85 | 3.18±0.78 | 3±0 |
|  | **Pyr** | 0.27±0.2 | 0.14±0.1 | 0.08±0.04 | 0.34±0.43 | 0.32±0.44 | 0.2±0.15 | 0.48±0.14 | 0.43±0.35 | 1.52±0.45 | 1.25±0.38 | 1.18±0.38 | 1.24±0.25 | 0.98±0.18 |
|  | **B(a)A** | 0.01±0 | <0.01 | <0.01 | 0.07±0 | <0.01 | <0.01 | 0.35±0 | <0.01 | 0.01±0 | 0.01±0.01 | <0.01 | 0.01±0 | <0.01 |
|  | **Cry** | <0.01 | <0.01 | <0.01 | <0.01 | <0.01 | <0.01 | <0.01 | <0.01 | <0.01 | <0.01 | <0.01 | <0.01 | <0.01 |
|  | **B (b)F** | <0.01 | <0.01 | <0.01 | <0.01 | <0.01 | <0.01 | <0.01 | <0.01 | <0.01 | <0.01 | <0.01 | <0.01 | <0.01 |
|  | **B(k)F** | <0.01 | <0.01 | <0.01 | <0.01 | <0.01 | <0.01 | <0.01 | <0.01 | <0.01 | <0.01 | <0.01 | <0.01 | <0.01 |
|  | **B(a)p** | <0.01 | <0.01 | <0.01 | <0.01 | <0.01 | <0.01 | <0.01 | <0.01 | <0.01 | <0.01 | <0.01 | <0.01 | <0.01 |
|  | **IP** | <0.01 | <0.01 | <0.01 | <0.01 | <0.01 | <0.01 | <0.01 | <0.01 | <0.01 | <0.01 | <0.01 | <0.01 | <0.01 |
|  | **D (a,h)A** | <0.01 | <0.01 | <0.01 | <0.01 | <0.01 | <0.01 | <0.01 | <0.01 | <0.01 | <0.01 | <0.01 | <0.01 | <0.01 |
|  | **B (ghi)P** | <0.01 | <0.01 | <0.01 | <0.01 | <0.01 | <0.01 | <0.01 | <0.01 | <0.01 | <0.01 | <0.01 | <0.01 | <0.01 |

** data derived from two measurements (additional sampling campaigns are scheduled for August and November 2022).*


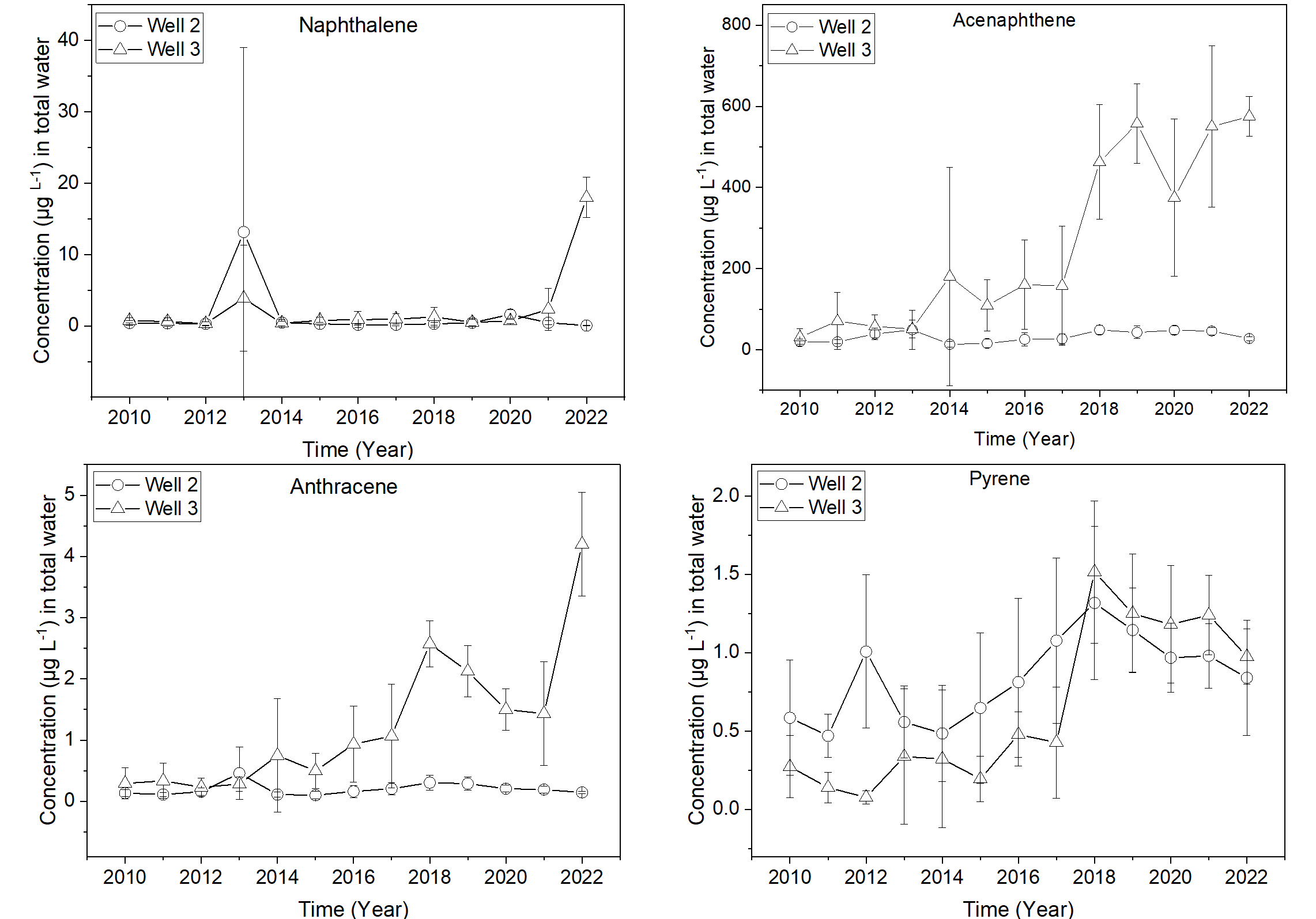


**Figure S2.** Temporal variability of individual PAH concentrations in total (i.e., unfiltered) groundwater samples from wells 2 and 3 based on conventional measurements (LMBV, 2022). For well 1, only ΣPAHs data was available.

**Table S7.** Estimated freely dissolved concentration C_w_ (L kg^-1^) in the LDPEs exposed to groundwater wells 1, 2 and 3 for two to six sampling times.

| Sampling well | Days | Nap | Acy | Ace | Flu | Phen | Ant | Flt | Pyr | B(a)A | Cry | B (b)F | B(k)F | B(a)p | IP | D (a,h)A | B (ghi)P |
| --- | --- | --- | --- | --- | --- | --- | --- | --- | --- | --- | --- | --- | --- | --- | --- | --- | --- |
| Well 1 | 42 | 0.03±0.01 | 0.18±0.08 | 0.18±0.04 | 0.12±0.02 | 0.14±0.01 | 0.06±0.01 | 0.35±0 | 0.16±0.01 | 0.001±0 | 0.001±0 | < 0.01 | < 0.01 | < 0.01 | < 0.01 | < 0.01 | < 0.01 |
|  | 80 | 0.05±0 | 0.19±0.14 | 0.51±0.58 | 0.43±0.31 | 0.06±0.02 | 0.04±0.02 | 0.51±0.11 | 0.25±0.04 | 0.002±0 | 0.004±0 | 0.0001±0 | 0.0001±0 | 0.0002±0 | < 0.01 | < 0.01 | < 0.01 |
| Well 2 | 42 | < 0.01 | 0.01±0 | 0.01±0 | < 0.01 | < 0.01 | 0.001±0 | 0.002±0 | 0.003±0 | < 0.01 | < 0.01 | < 0.01 | < 0.01 | < 0.01 | < 0.01 | < 0.01 | < 0.01 |
|  | 80 | nd | 0.01±0 | 0.03±0.02 | 0.01±0 | nd | 0.002±0 | 0.01±0 | 0.01±0 | nd | 0.0001±0 | nd | nd | nd | nd | nd | nd |
| Well 3 | 14 | 0.14±0.01 | 0.38±0.04 | 27.62±5.89 | 0.39±0.56 | 0.05±0.04 | 0.01±0 | 0.01±0 | 0.003±0 | < 0.01 | < 0.01 | < 0.01 | < 0.01 | < 0.01 | < 0.01 | < 0.01 | < 0.01 |
|  | 28 | 0.06±0.03 | 0.35±0.03 | 28.91±2.12 | 0.82±0.14 | 0.04±0 | 0.01±0.01 | 0.01±0 | 0.004±0 | < 0.01 | < 0.01 | < 0.01 | < 0.01 | < 0.01 | < 0.01 | < 0.01 | < 0.01 |
|  | 42 | 0.04±0.01 | 0.35±0.02 | 27.21±2.41 | 0.67±0.14 | 0.03±0.01 | 0.01±0 | 0.01±0 | 0.005±0 | < 0.01 | < 0.01 | < 0.01 | < 0.01 | < 0.01 | < 0.01 | < 0.01 | < 0.01 |
|  | 56 | 0.04±0.01 | 0.3±0.06 | 26.36±3.83 | 0.56±0.19 | 0.04±0.01 | 0.02±0.01 | 0.01±0 | 0.01±0 | < 0.01 | < 0.01 | < 0.01 | < 0.01 | < 0.01 | < 0.01 | < 0.01 | < 0.01 |
|  | 70 | 0.06±0.02 | 0.29±0.05 | 28.1±5.16 | 0.55±0.28 | 0.04±0.01 | 0.03±0.01 | 0.01±0 | 0.01±0 | < 0.01 | < 0.01 | < 0.01 | < 0.01 | < 0.01 | < 0.01 | < 0.01 | < 0.01 |
|  | 80 | 0.05±0.01 | 0.28±0.01 | 28.32±1.08 | 0.45±0.16 | 0.03±0.01 | 0.03±0.01 | 0.02±0 | 0.01±0 | nd | nd | nd | nd | nd | nd | nd | nd |


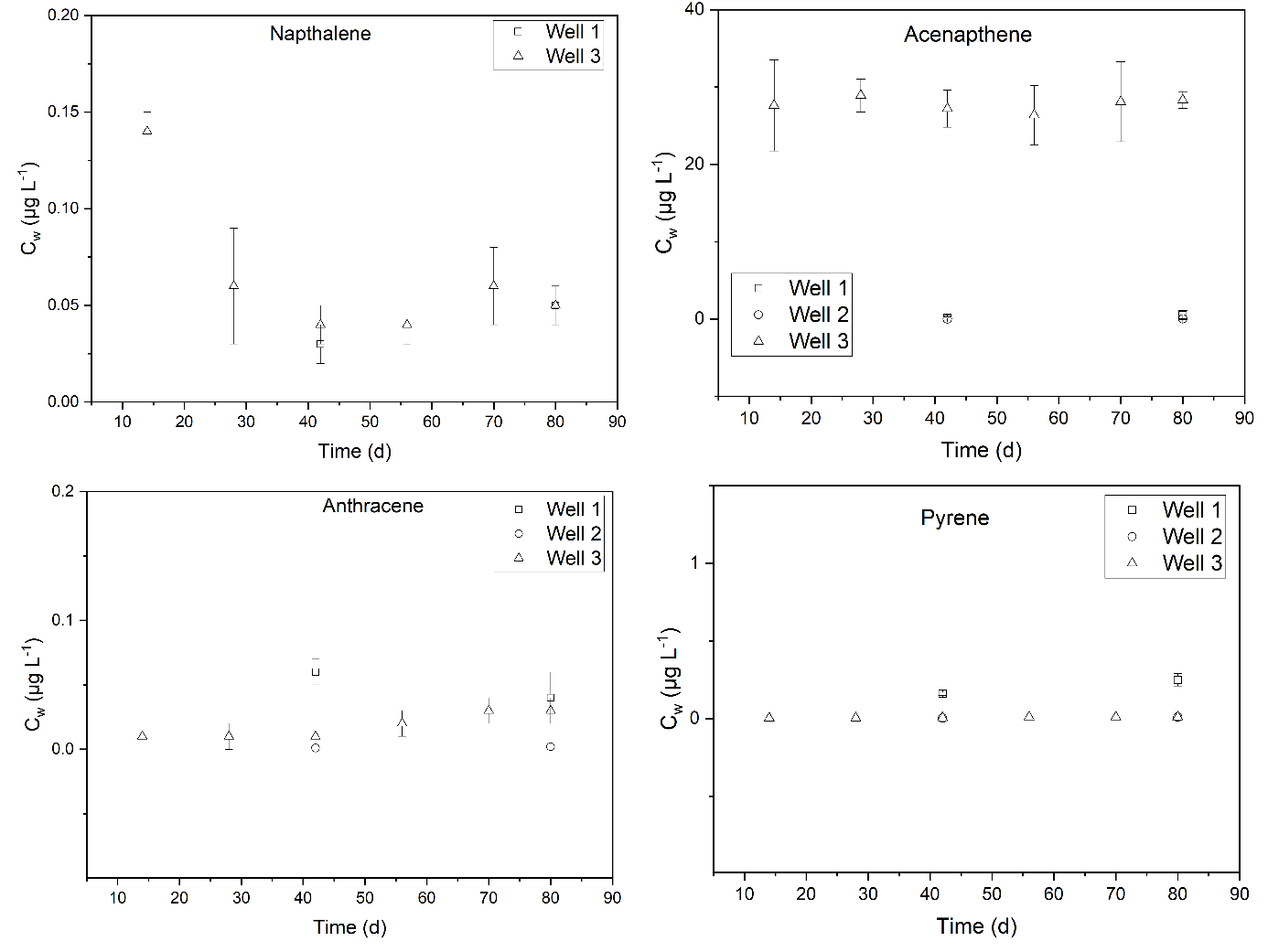


**Figure S3.** Temporal variability of freely dissolved concentrations (C_w_) of individual PAHs measured at groundwater wells 1, 2 and 3. C_w_ was measured six times at well 3 and twice at wells 1 and 2.

**Table S8.** PRC retained by the LDPE, *f_ret_*, over the course of the deployment.

| Sampling well | Day | d_10_-ANT | d_10_-FLUO | d_12_-BaA | d_12_-BaP | d_14_-DBahA |
| --- | --- | --- | --- | --- | --- | --- |
|  |  |  |  |  |  |  |
| Well 1 | 42 | 0.06 | 0.15 | 0.79 | 0.89 | 0.70 |
|  | 80 | 0 | 0.09 | 0.68 | 0.62 | 0.59 |
| Well 2 | 42 | 0 | 0.00 | 0.19 | 0.91 | 0.86 |
|  | 80 | 0 | 0.00 | 0.03 | 0.63 | 0.63 |
| Well 3 | 14 | 0.45 | 0.59 | 0.69 | 0.74 | 0.70 |
|  | 28 | 0.3 | 0.56 | 0.66 | 0.72 | 0.66 |
|  | 42 | 0.22 | 0.48 | 0.64 | 0.67 | 0.64 |
|  | 56 | 0.15 | 0.47 | 0.66 | 0.60 | 0.64 |
|  | 70 | 0.11 | 0.36 | 0.6 | 0.57 | 0.58 |
|  | 80 | 0.08 | 0.32 | 0.64 | 0.54 | 0.55 |


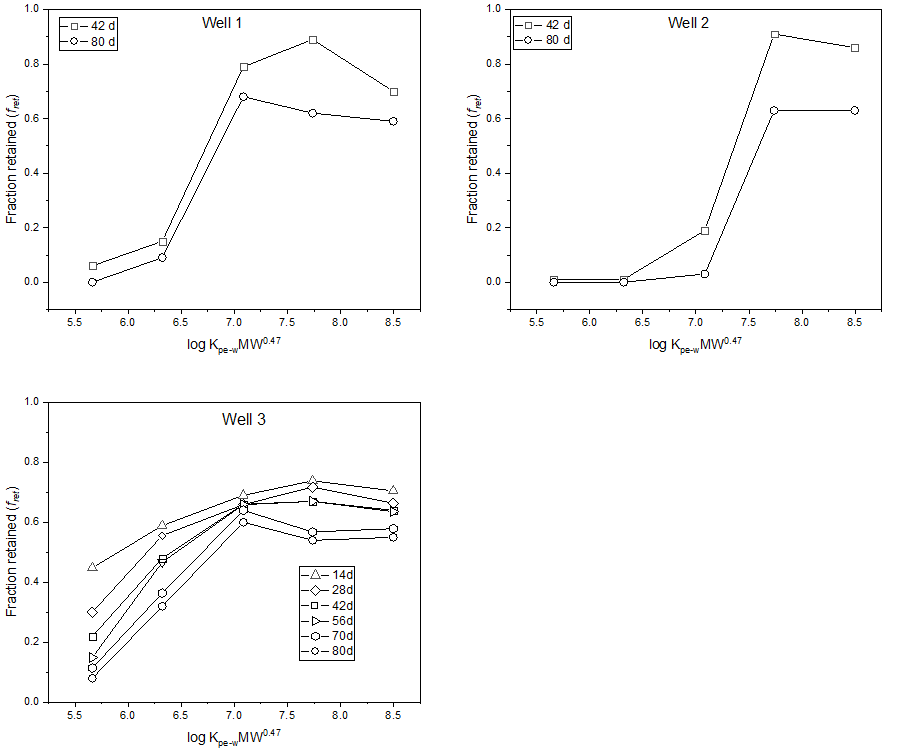


**Figure S4.** Fraction of retained PRCs (f_ret_) as a function of log K_PE-W_ MW^0.47^ for groundwater wells 1, 2 and 3.


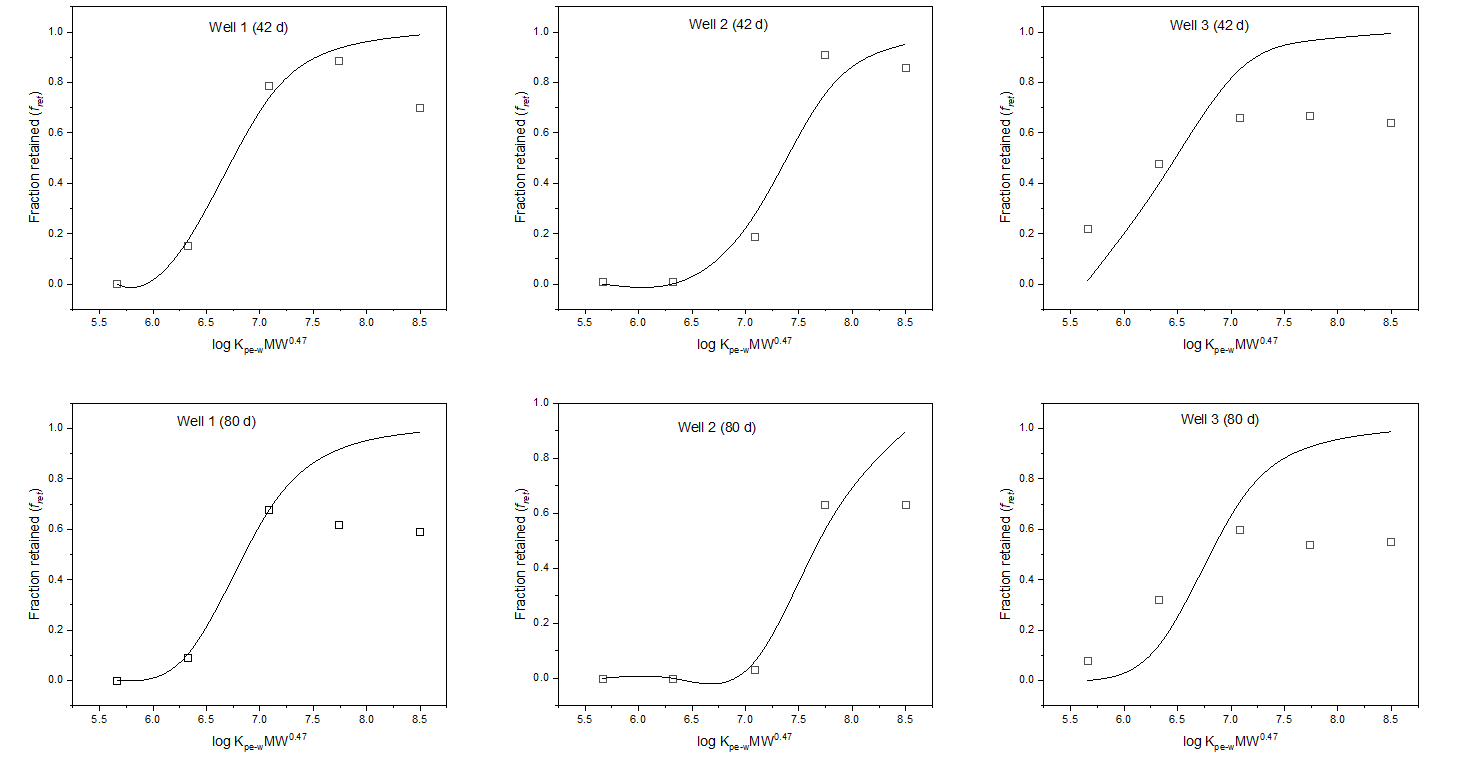


**Figure S5.** Fraction of retained PRCs (f_ret_) and model fit for 42 d and 80 d exposure period. The drawn regression lines represent the model fit.


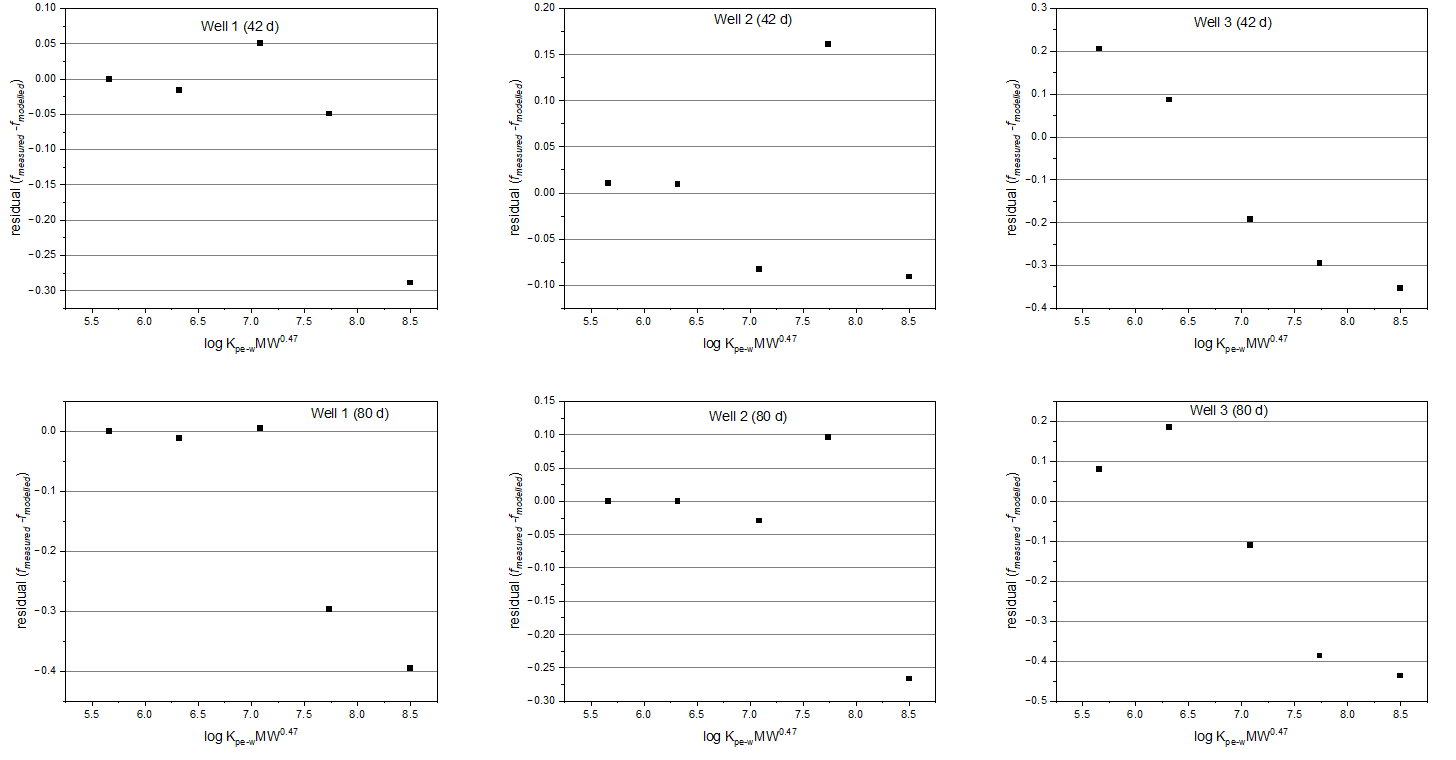


**Figure S6.** Residuals of the fraction of retained PRCs (f_ret_) for 42 d and 80 d exposure period

**Table S9.** The degree of equilibrium (DEQ) attained during the exposure period at the investigated groundwater wells. A value of ‘1’ reflects complete equilibrium.

| Sampling well | Day | Nap | Acy | Ace | Flu | Phen | Ant | Flt | Pyr | B(a)A | Cry | B (b)F | B(k)F | B(a)p | IP | D (a,h)A | B (ghi)P |
| --- | --- | --- | --- | --- | --- | --- | --- | --- | --- | --- | --- | --- | --- | --- | --- | --- | --- |
|  |  |  |  |  |  |  |  |  |  |  |  |  |  |  |  |  |  |
| Well 1 | 42 | 1 | 1 | 1 | 1 | 1 | 1 | 0.75 | 0.69 | 0.27 | 0.28 | 0.08 | 0.06 | 0.05 | 0.02 | 0.02 | 0.02 |
|  | 80 | 1 | 1 | 1 | 1 | 1 | 1 | 0.83 | 0.78 | 0.33 | 0.34 | 0.11 | 0.08 | 0.07 | 0.02 | 0.02 | 0.02 |
| Well 2 | 42 | 1 | 1 | 1 | 1 | 1 | 1 | 1 | 0.99 | 0.73 | 0.75 | 0.31 | 0.24 | 0.21 | 0.06 | 0.07 | 0.06 |
|  | 80 | 1 | 1 | 1 | 1 | 1 | 1 | 1 | 1 | 0.94 | 0.95 | 0.55 | 0.45 | 0.41 | 0.13 | 0.14 | 0.13 |
| Well 3 | 14 | 1 | 1 | 1 | 0.97 | 0.68 | 0.59 | 0.18 | 0.16 | 0.04 | 0.05 | 0.01 | 0.01 | 0.01 | 0 | 0 | 0 |
|  | 28 | 1 | 1 | 1 | 1 | 0.87 | 0.8 | 0.31 | 0.27 | 0.08 | 0.08 | 0.02 | 0.02 | 0.01 | 0 | 0 | 0 |
|  | 42 | 1 | 1 | 1 | 1 | 0.98 | 0.96 | 0.52 | 0.46 | 0.15 | 0.16 | 0.04 | 0.03 | 0.03 | 0.01 | 0.01 | 0.01 |
|  | 56 | 1 | 1 | 1 | 1 | 0.99 | 0.96 | 0.53 | 0.48 | 0.15 | 0.16 | 0.05 | 0.03 | 0.03 | 0.01 | 0.01 | 0.01 |
|  | 70 | 1 | 1 | 1 | 1 | 1 | 1 | 0.76 | 0.7 | 0.27 | 0.28 | 0.08 | 0.06 | 0.06 | 0.02 | 0.02 | 0.02 |
|  | 80 | 1 | 1 | 1 | 1 | 1 | 1 | 0.79 | 0.74 | 0.29 | 0.31 | 0.09 | 0.07 | 0.06 | 0.02 | 0.02 | 0.02 |

**Table S10.** Estimates of water boundary layer mass transfer coefficient *(k_w_),* water boundary layer thickness *(*δ_w_), and analyte uptake control assessment ( *I_p_/I_w_ )*.

| PAH | Well 1 | | | Well 2 | | | Well 3 | | |
| --- | --- | --- | --- | --- | --- | --- | --- | --- | --- |
|  | *k_w_*  (µm s^-1^ ) | δ_w_  (µm) | *I_p_/I_w_*  *_(_*_unitless_*_)_* | *k_w_*  (µm s^-1^ ) | δ_w_  (µm) | *I_p_/I_w_*  *_(_*_unitless_*_)_* | *k_w_*  (µm s^-1^ ) | δ_w_  (µm) | *I_p_/I_w_*  *_(_*_unitless_*_)_* |
| Nap | 5.82 | 147.51 | 0.09 | 41.76 | 20.54 | 0.65 | 5.08 | 168.96 | 0.08 |
| Acy | 5.36 | 141.55 | 0.05 | 38.53 | 19.71 | 0.37 | 4.68 | 162.14 | 0.04 |
| Ace | 5.33 | 141.11 | 0.04 | 38.29 | 19.65 | 0.28 | 4.66 | 161.63 | 0.03 |
| Flu | 5.15 | 138.60 | 0.04 | 36.96 | 19.30 | 0.26 | 4.49 | 158.75 | 0.03 |
| Phen | 4.98 | 136.30 | 0.02 | 35.77 | 18.98 | 0.11 | 4.35 | 156.12 | 0.01 |
| Ant | 4.98 | 136.30 | 0.01 | 35.77 | 18.98 | 0.09 | 4.35 | 156.12 | 0.01 |
| Flt | 4.69 | 132.21 | 0.00 | 33.70 | 18.41 | 0.03 | 4.10 | 151.44 | 0.00 |
| Pyr | 4.69 | 132.21 | 0.00 | 33.70 | 18.41 | 0.03 | 4.10 | 151.44 | 0.00 |
| B(a)A | 4.43 | 128.43 | 0.00 | 31.84 | 17.88 | 0.02 | 3.87 | 147.11 | 0.00 |
| Cry | 4.43 | 128.43 | 0.00 | 31.84 | 17.88 | 0.02 | 3.87 | 147.11 | 0.00 |
| B (b)F | 4.23 | 125.38 | 0.00 | 30.38 | 17.46 | 0.01 | 3.69 | 143.62 | 0.00 |
| B(k)F | 4.23 | 125.38 | 0.00 | 30.38 | 17.46 | 0.01 | 3.69 | 143.62 | 0.00 |
| B(a)p | 4.23 | 125.38 | 0.00 | 30.38 | 17.46 | 0.00 | 3.69 | 143.62 | 0.00 |
| IP | 4.05 | 122.68 | 0.00 | 29.11 | 17.08 | 0.00 | 3.54 | 140.52 | 0.00 |
| D (a,h)A | 4.04 | 122.47 | 0.00 | 29.01 | 17.05 | 0.00 | 3.53 | 140.28 | 0.00 |
| B (ghi)P | 4.05 | 122.68 | 0.00 | 29.11 | 17.08 | 0.00 | 3.54 | 140.52 | 0.00 |

**References**

1. Abraham, M.H., McGowan, J.C., 1987. The use of characteristic volumes to measure cavity terms in reversed phase liquid chromatography. Chromatographia 23 (4), 243–246.
2. Booij, K., Smedes, F., Allan, I.J., 2017. Guidelines for determining polymer-water and polymer-polymer partition coefficients of organic compounds.
3. Jonker, M.T.O., 2016. Determining octanol–water partition coefficients for extremely hydrophobic chemicals by combining “slow stirring” and solid‐phase microextraction. Environmental Toxicology and Chemistry: An International Journal 35 (6), 1371–1377.
4. Lohmann, R., 2012. Critical review of low-density polyethylene’s partitioning and diffusion coefficients for trace organic contaminants and implications for its use as a passive sampler. Environmental Science & Technology 46 (2), 606–618.
5. Ma, Y.-G., Lei, Y.D., Xiao, H., Wania, F., Wang, W.-H., 2010. Critical review and recommended values for the physical-chemical property data of 15 polycyclic aromatic hydrocarbons at 25 C. Journal of Chemical & Engineering Data 55 (2), 819–825.
